# Wind‐Induced Variability of Warm Water on the Southern Bellingshausen Sea Continental Shelf

**DOI:** 10.1029/2022JC018636

**Published:** 2022-11-08

**Authors:** Ria Oelerich, Karen J. Heywood, Gillian M. Damerell, Andrew F. Thompson

**Affiliations:** ^1^ Centre for Ocean and Atmospheric Sciences School of Environmental Sciences University of East Anglia Norwich UK; ^2^ Environmental Science and Engineering California Institute of Technology Pasadena CA USA

**Keywords:** GLORYS12V1 reanalysis, Bellingshausen Sea, heat transport, frontal jet, cold water formation, Amundsen Sea Low

## Abstract

The Bellingshausen Sea hosts heat transport onto the continental shelf, potentially enhancing ice shelf basal melt. Here, we use the GLORYS12V1 1993–2018 reanalysis to identify physical processes that set seasonal and interannual variability of water mass properties in the Eltanin and Latady Bays on the southern Bellingshausen Sea continental shelf. Annual means of potential temperature from 300 m to the seabed reveal interannual variability and allow separation into warm and cold regimes. The Amundsen Sea Low (ASL) is more intense and extends further east during the warm regime than the cold regime. In the warm regime, a wind‐induced reduction of sea ice concentration near the coast increases surface heat loss, convection, and formation of cold dense water in winter, associated with a decrease in heat content of the southern Bellingshausen Sea over time and a net northward heat transport. In contrast, in the cold regime, increased sea ice concentration reduces surface heat loss and thus formation of cold, dense water. Combined with an increase in heat content over time and a net southward heat transport, this results in a warming of the southern Bellingshausen Sea. This suggests that variability in the deep water temperature in the southern Bellingshausen Sea is primarily due to local surface heat fluxes above the shelf. The variability of surface heat fluxes is related to the variability of the ASL and its influence on sea ice extent and local formation of cold, dense water in winter.

## Key Points

- Intensity and location of Amundsen Sea Low (ASL) affect ocean properties of southern Bellingshausen Sea continental shelf in 26‐year reanalysis
- The ASL modifies the surface heat flux, warming Bellingshausen Sea shelf when it shifts westward and weakens, and vice versa
- The southern Bellingshausen Sea temperature depends more on surface heat fluxes above the continental shelf than southward heat transport

## 1 Introduction

**Figure 1:**
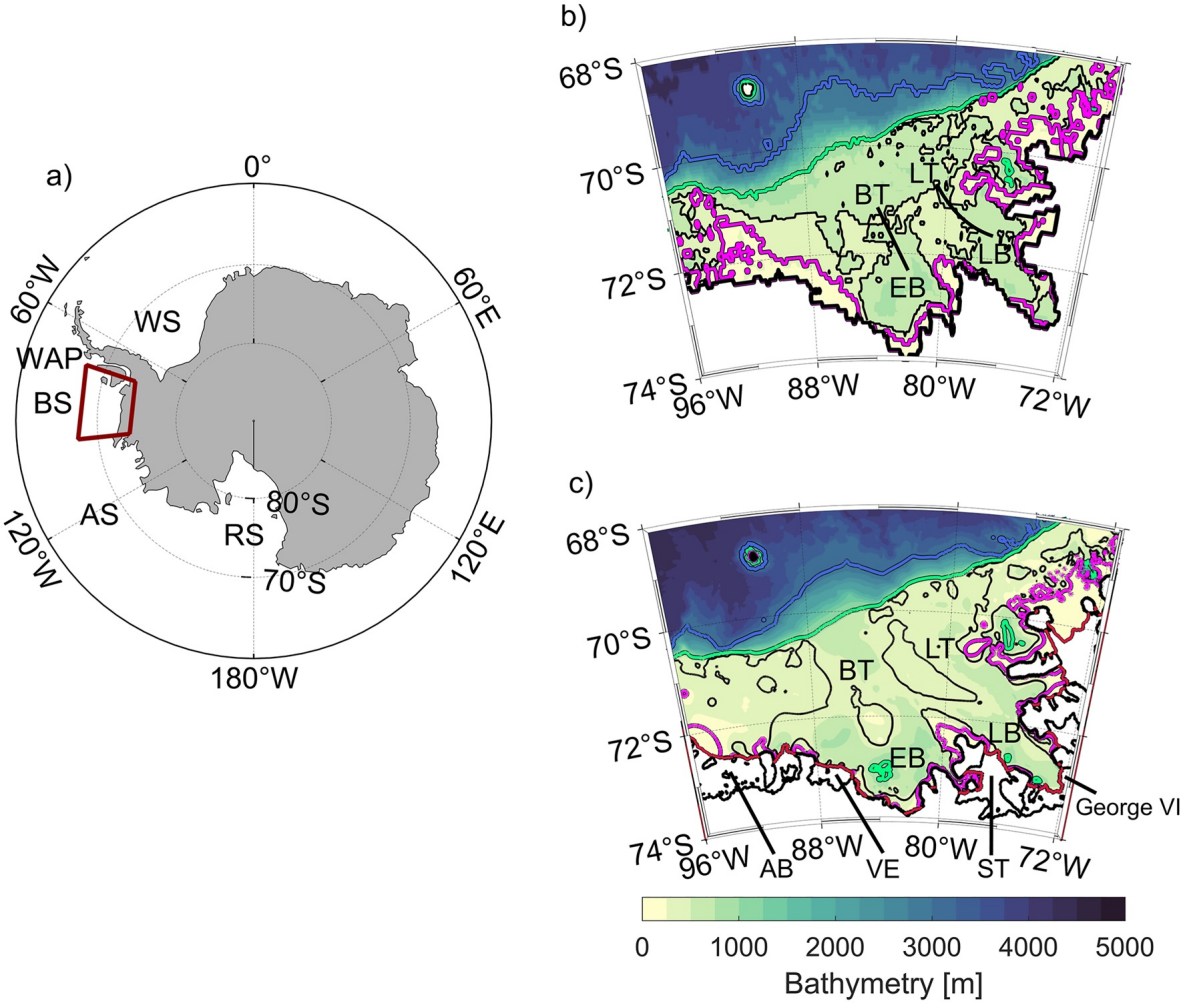
(a) Map of the Southern Ocean, with the study region outlined by a red box (b) bathymetry of the Bellingshausen Sea (red box in (a)) extracted from GLORYS (GEBCO8) with black lines indicating the Belgica and Latady Trough locations, and (c) bathymetry of the Bellingshausen Sea extracted from the R‐Topo2 data product (Schaffer et al., 2016). The colored contours indicate the 3,000‐m isobath (blue), the 1,000‐m isobath (green), the 500‐m isobath (black), and the 300‐m isobath (magenta) with the coastline (bold black). For the purposes of this study, the shelf break is defined as the 1,000‐m isobath. Ice shelves along the coast are indicated by red contours. Key geographic features in (a) and (b) are labeled: Bellingshausen Sea (BS), Amundsen Sea (AS), West Antarctic Peninsula (WAP), Ross Sea (RS), Weddell Sea (WS), Belgica Trough (BT), Latady Trough (LT), Eltanin Bay (EB), Latady Bay (LB), Abbot Ice Shelf (AB), Venable Ice Shelf (VE), Stange Ice Shelf (ST), Wilkins Ice Shelf (WI), and George VI Ice Shelf (George VI).

The major oceanic source of heat onto the Antarctic continental shelf is Circumpolar Deep Water (CDW) circulating eastward within the Antarctic Circumpolar Current (ACC). Surface heat and freshwater fluxes can also make a significant contribution to regional budgets and modify stratification that also feeds back on the geostrophic circulation and associated tracer transport (Couto et al., 2017). The southernmost part of the ACC is generally considered to play an important role in bringing warm water onto the continental shelf particularly in the West Antarctic shelf seas (e.g., Dinniman & Klinck, 2004; Jenkins & Jacobs, 2008; Martinson & McKee, 2012). Here, we consider the Bellingshausen Sea (Figure 1), where the ACC approaches most closely to the shelf break (e.g., Jenkins & Jacobs, 2008; Schulze‐Chretien et al., 2021; Thompson et al., 2020).

CDW is the main water mass that has been associated with the thinning of West Antarctic ice shelves through basal melt (e.g., Cook et al., 2016; Paolo et al., 2015). Troughs on the continental shelf of Antarctica provide direct routes for CDW, or slightly colder modified CDW (mCDW), to gain access to coastal regions (Jacobs et al., 2011; Wåhlin et al., 2010). The Bellingshausen Sea lies between two more well‐studied regions, the Amundsen Sea to the west and the West Antarctic Peninsula (WAP) to the east, where unmodified CDW (*θ* > 1.5 °C) can access the deeper troughs on the shelf (e.g., Dotto et al., 2019; Jenkins & Jacobs, 2008; Martinson & McKee, 2012). Shoreward transport across the shelf can enable mCDW (*θ* < 1.5 °C) to reach deep cavities below the ice shelves inducing a retreat of their grounding lines (Dutrieux et al., 2014; Jenkins et al., 2010; Konrad et al., 2018).

Most of the ice shelves along the coast of the southern Bellingshausen Sea have experienced a significant loss in volume and increased basal melt rates over the past decades (Hogg et al., 2017; Paolo et al., 2015; Rignot et al., 2019), and ocean forcing has been implicated. Jenkins and Jacobs (2008) observed mCDW with a temperature warmer than 1 °C flooding the Bellingshausen Sea continental shelf and an inflow of warm water into the ice shelf cavities. Schulze‐Chretien et al. (2021) analyzed ship‐based observations and showed that submarine troughs provide topographically steered pathways for mCDW from the shelf break toward the deep embayments close to the floating ice shelves. mCDW enters the continental shelf through the eastern side of one of the Bellingshausen Sea's major troughs, the Belgica Trough (Figure 1c). Seal‐acquired observations have indicated a cyclonic circulation within this trough with flow extending toward the coast along its eastern boundaries and returning to the shelf break along western boundaries (Zhang et al., 2016). Model studies and seal‐acquired observations have provided evidence for a coherent westward current along the Bellingshausen Sea coastline, the Antarctic Coastal Current acting as a link between the WAP and the Amundsen Sea (Holland et al., 2010; Schubert et al., 2021).

Around much of Antarctica, the Antarctic Slope Current (ASC) provides westward transport along the continental slope. Beneath it, the Antarctic Slope Undercurrent, a bottom‐intensified current, flows eastward (Chavanne et al., 2010). The formation and persistence of the ASC are mostly attributed to surface wind stress, and the intensity and variability of the ASC largely control the rate at which heat associated with CDW moves across the slope and onto the continental shelf (Thompson et al., 2018). Wind‐driven variations in intensity of the Antarctic Slope Undercurrent have been suggested as a mechanism for the transport of heat into major troughs of the Amundsen Sea (Dotto et al., 2020; Walker et al., 2013).

In contrast, the ASC is absent in the central and eastern Bellingshausen Sea (Thompson et al., 2018), as it is in the WAP. There are insufficient observations to determine if the Antarctic Slope Undercurrent is a persistent feature in this section of the Antarctic continental shelf (Thompson et al., 2018). These arguments suggest that mechanisms for heat transport onto the continental shelf may differ from those of the Amundsen Sea. The eastward flow marking the southern limit of the ACC (Thompson et al., 2020) is found in close proximity to the shelf break in the Bellingshausen Sea and WAP allowing CDW unhindered access to the continental shelf (e.g., Graham et al., 2016; Martinson et al., 2008; Nakayama et al., 2018; Smith et al., 1999). However, it remains unclear whether variations in location and intensity of the frontal jet are associated with variations in the regional wind patterns and whether the frontal jet influences the heat transported onto the Bellingshausen Sea continental shelf and toward the ice shelf cavities. At the western limit of the Bellingshausen Sea, there is evidence for a bottom‐intensified ASC above the slope (Nakayama et al., 2018; Thompson et al., 2020; Zhang et al., 2016). Thompson et al. (2020) identified an eastward current at the western limit of the Bellingshausen Sea (west of the Belgica Trough) located between a shallow westward flow of surface waters and a deeper westward flow that extends from 1,500 m to the seafloor. This eastward current is associated with the shoreward extent of offshore CDW and is suggested to be the source of warm water entering the Belgica Trough (Thompson et al., 2020).

Wind fields in the West Antarctic sector are dominated by a low‐pressure system, the Amundsen Sea Low (ASL), centered in the Amundsen Sea (Hosking et al., 2013, 2016). Many studies have suggested that variations in the CDW inflow to the Amundsen Sea are linked to the wind field above the continental shelf break (e.g., Kim et al., 2017; Steig et al., 2012; Thoma et al., 2008). Dinniman et al. (2012) demonstrated with a regional ocean‐sea ice‐ice shelf simulation that the inflow of CDW onto the WAP shelf is dependent on both wind strength and ACC transport. Due to the positioning of the ASL, eastward winds occur above the shelf break and slope that directly control the cross‐slope heat flux through current fluctuations within both major troughs, the Getz‐Dotson and the Pine Island‐Thwaites Troughs (e.g., Assmann et al., 2013; Thoma et al., 2008; Wåhlin et al., 2013). Ekman pumping associated with the regional wind pattern is another possible mechanism to deliver warm waters onto the continental shelf (e.g., Assmann et al., 2019; Kim et al., 2017; Kimura et al., 2017). Similarly, Martinson et al. (2008) suggested wind‐driven upwelling of offshore CDW as a possible mechanism for the delivery of CDW onto the WAP shelf. However, unlike the Amundsen Sea, the winds associated with the ASL in the Bellingshausen Sea tend to be onshore rather than alongshore, so this region experiences the weakest along‐slope winds of the Antarctic margins (Hazel & Stewart, 2019; Turner et al., 2013). Modeling studies and observations at the WAP indicate that when the mean shelf break flow encounters curving bathymetry, some of the water within the ACC is carried onto the shelf by momentum, if the forcing is strong enough (Dinniman & Klinck, 2004; Klinck et al., 2004). At the WAP, eddy heat fluxes at the shelf break were also identified as a mechanism for on‐shelf heat transport (Couto et al., 2017). Conditions that are favorable for mCDW to access the continental shelf and to reach the southern Bellingshausen Sea are still uncertain, largely due to the lack of long‐term observations in this particularly inaccessible area.

Although much of the literature concerning interannual variability in heat content over the West Antarctic continental shelf has focused on shelf break processes, other studies have highlighted processes local to the shelf. St‐Laurent et al. (2015) and Webber et al. (2017) emphasized the influence of local sea ice formation and air‐sea heat fluxes on water temperatures over the Amundsen Sea continental shelf. Additionally, Kim et al. (2021) found much larger variability in mCDW properties in the Dotson Trough close to the coast, as compared to at the shelf break. Warm and cold regimes in the Amundsen Sea have been identified using observations (e.g., Jenkins et al., 2018; Webber et al., 2017) and models (e.g., Dotto et al., 2019; Dutrieux et al., 2014; Nakayama et al., 2018). However, the lack of long‐term observations on the continental shelf of the Bellingshausen Sea means that interannual temperature variability there has not yet been studied.

In this study, we identify conditions and processes related to warm and cold regimes on the continental shelf of the Bellingshausen Sea, particularly within Eltanin and Latady Bays near the southern coast using a high‐resolution global ocean reanalysis. We have been unable to find a name in the literature for the large bay at the southern end of the Latady Trough (Figure 1), and will therefore refer to it as Latady Bay. We test the hypothesis that changes of the ASL's location and intensity in the Bellingshausen Sea determine the ocean conditions on the continental shelf. We use the GLORYS12V1 reanalysis first to describe spatial and temporal variability of temperature, heat content, and surface heat fluxes, second to identify conditions that represent warm and cold conditions in the Bellingshausen Sea, and third to identify processes that are responsible for warming and cooling. The paper is organized as follows: Section 2 introduces and describes the GLORYS12V1 reanalysis in the Bellingshausen Sea. Section 3 quantifies and discusses spatial and temporal variability. Section 4 presents warm and cold regimes using composites and anomalies with respect to the long‐term mean, and discusses processes driving warming or cooling with comparison to previous studies. Section 5 summarizes the main conclusions and offers suggestions for future work.

## 2 The GLORYS12V1 Reanalysis and Climatology

The GLORYS12V1 reanalysis, hereinafter referred to as GLORYS, provided by the Copernicus Marine Environment Monitoring Service (CMEMS), is a global ocean product with a horizontal resolution of $\frac{1}{12}$° (∼3 km in the Bellingshausen Sea) and 50 vertical z‐levels covering the altimetry era from 1993 onward (Fernandez & Lellouche, 2021). Typically, most recent products are available with a 24‐month delay. This study uses output up to December 2018. GLORYS assimilates sea level anomalies of all altimetric satellites, potential temperature and practical salinity profiles from the CMEMS CORAv4.1 database and sea ice concentration from the satellite data processing and distribution center of Ifremer (CERSAT database, https://cersat.ifremer.fr/). The implemented sea ice model is LIM2 and fully coupled to the ocean. Climatological river run‐off based on Dai et al. (2009) and freshwater fluxes from icebergs for Antarctica are implemented. The GLORYS ocean model component is the NEMO platform driven at the surface by ERA‐Interim atmospheric forcing. In this study, we show wind fields extracted from the fifth generation of the European Center for Medium‐Range Weather Forecasts (ECWMF) global climate reanalysis, ERA5 (Hersbach et al., 2019) that reflects the same patterns of variability as its predecessor ERA‐Interim within the considered time period in the Bellingshausen Sea, but with much higher horizontal resolution (0.36° versus ERA‐Interim 1°). The model bathymetry is based on ETOPO1 for the deep ocean and GEBCO8 for the coast and continental shelf (Figure 1b) and does not include ice shelf cavities (discussed further below).

**Figure 2:**
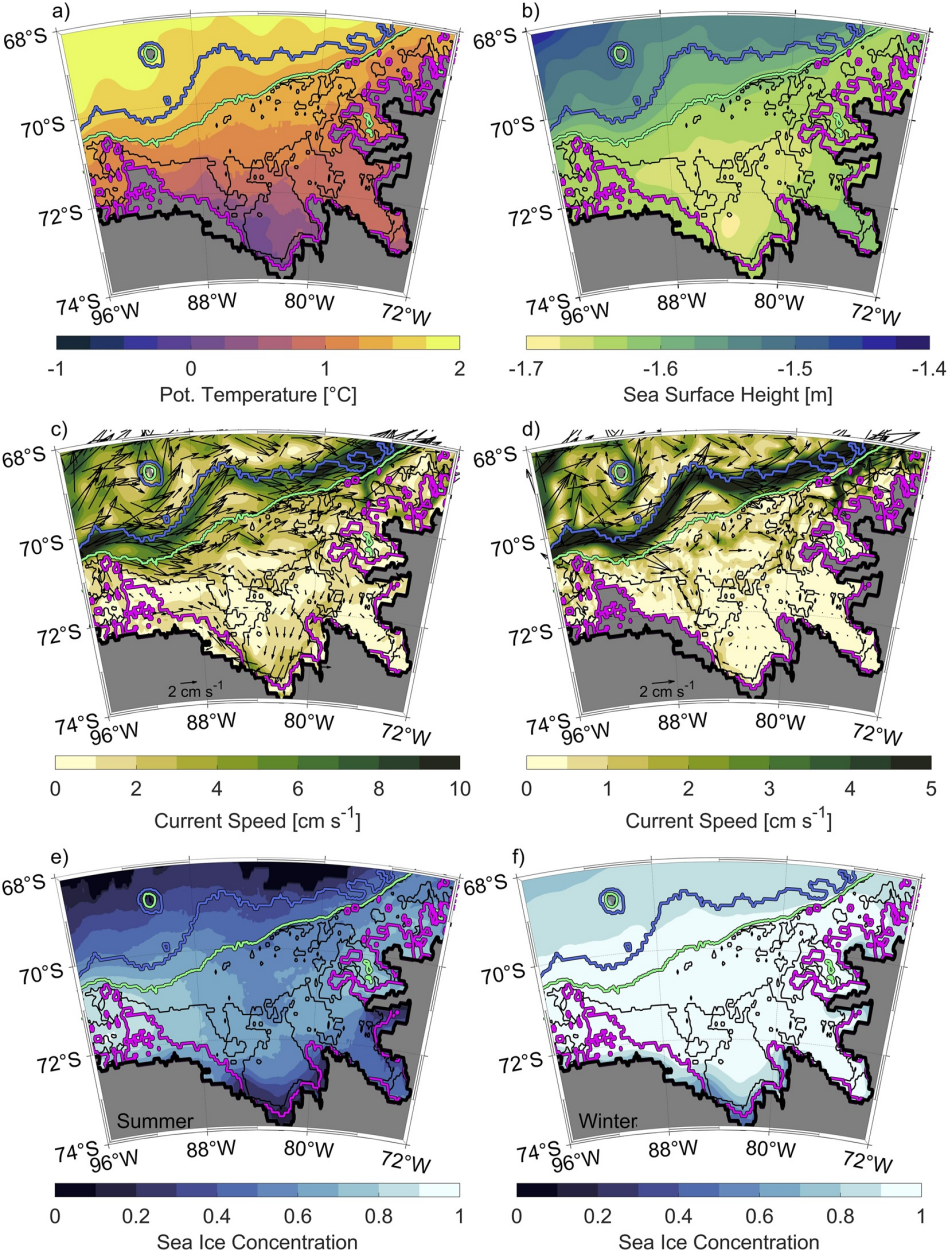
Long‐term mean from 1993 to 2018 of (a) potential temperature below 300 m, (b) sea surface height (SSH), (c) current speed above 300 m superimposed with velocity arrows, (d) current speed below 300 m superimposed with velocity arrows, (e) summer sea ice concentration, and (f) winter sea ice concentration, all extracted from the GLORYS reanalysis. Note the different scales used in panels (c) and (d). Isobaths are shown as in Figure 1.

Regional models with higher resolution and a more detailed bathymetry, providing a more accurate representation of the major troughs than in GLORYS, have been used to simulate the Bellingshausen Sea area (Flexas et al., 2022; Graham et al., 2016; Nakayama et al., 2018). Moreover, katabatic winds driving the sea ice away from coastal regions (e.g., coastal polynya region) may occur in the southern Bellingshausen Sea. Due to the sporadic and short‐lived occurrence of katabatic winds, they are not well captured in the monthly mean and annual mean analysis provided in this study. Katabatic winds are thus not considered in detail. Nonetheless, using a global reanalysis such as GLORYS has the advantage that it captures the response to larger‐scale temporal and atmospheric variability better than regional high‐resolution models. Therefore, we find GLORYS best suited to investigate temporal and spatial variability as it assimilates all available data and provides a continuous time series of 26 years, longer than available for most regional models. Here, we use annual and monthly means of potential temperature, sea surface height (SSH), current velocities, and sea ice concentration in combination with wind velocity and wind stress curl from 1993 to 2018 in the Bellingshausen Sea domain (Figure 1). We take the means of these parameters over the whole time period from 1993 to 2018 as representative of the long‐term mean state (Figure 2).

**Figure 3:**
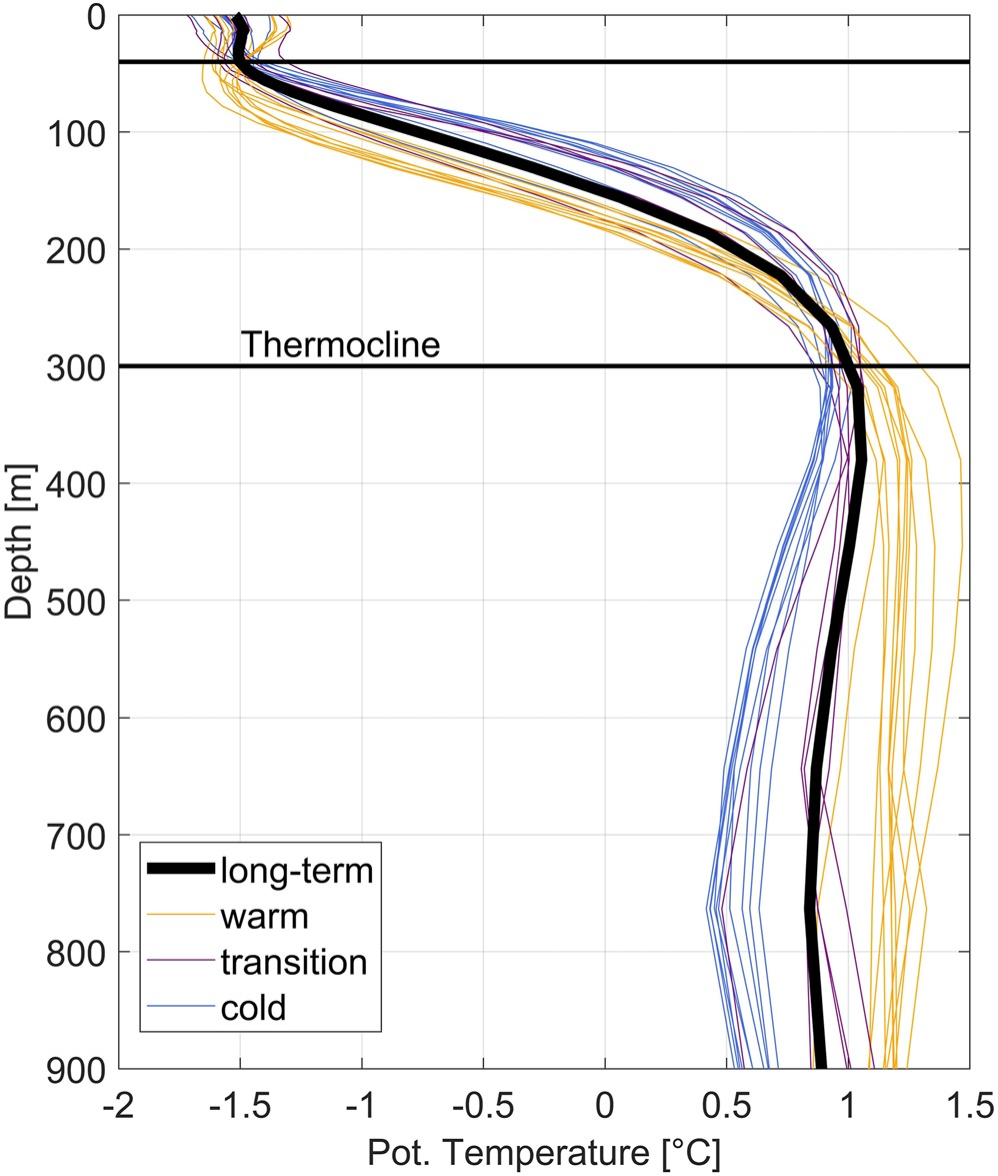
Vertical profiles of potential temperature averaged over all on‐shelf grid points (i.e., points shallower than 1,000 m). Annual average profiles for the warm years (1993–1995 and 2008–2015, orange lines), cold years (1997–2006, blue lines), and transition years (1996, 2007 and 2016–2018, purple lines). The warm, cold, and transition years are defined as in Section 3. The long‐term mean vertical profile is shown as a thick black line. Horizontal black lines indicate top and bottom of the average thermocline with the bottom of the thermocline located at a depth of 300 m on average.

The main focus of this study is the properties of mCDW (*θ* < 1.5 °C), which is the water mass most likely to enter ice shelf cavities and contribute heat to the melting process (e.g., Assmann et al., 2019; Jacobs et al., 2012; Kimura et al., 2017). The average depth of the thermocline, the upper boundary of mCDW, over space and time on the Bellingshausen Sea continental shelf is ∼300 m (Figure 3). Spatially averaged vertical profiles (Figure 3) indicate variations of the thermocline depth from 270 to 370 m associated with bottom temperature variations. Bottom temperature variations indicate two regimes, a cold regime and a warm regime. In the cold regime, the bottom temperatures of the average vertical profile (Figure 3) are colder than average and the thermocline is shallower. Likewise, if the bottom temperatures of a vertical profile are warmer than average the thermocline is deeper. In order to capture the temperature variations in lower levels of the water column, we use vertically averaged temperatures from 300 to 1,000 m (or to the seabed for shallower areas) for spatial and temporal analysis. Hereinafter, all vertical averages imply “below 300 m” unless stated otherwise. The results are similar if the average is calculated using the water column below the temperature maximum, so the latter is not presented here. We also tested using the mCDW layer thickness which has been demonstrated to be the main driver in heat content variability on the Amundsen Sea continental shelf (e.g., Jenkins et al., 2018; Kim et al., 2021; Thoma et al., 2008), but this approach gave similar results to the average temperature below 300 m, so is not presented here. In general, GLORYS has small regional temperature biases (<0.4 °C) in temperature with respect to the Worlds Ocean Atlas climatology 2013 and in situ data (Drévillon et al., 2021). Drévillon et al. (2021) further stated that the largest biases of up to 0.4 °C may occur in the 50–100 m layer and in the northern Atlantic and Southern Ocean. In this study, our analysis is mainly focused on vertically averaged temperatures below 300 m which minimizes the possibility of biases as much as possible.

The frontal jet (Figures 2c and 2d), representing the ACC's southern boundary, occurs between the shelf break (1,000‐m isobath) and the 3,000‐m isobath. Its distance to the shelf break varies along the continental slope of the Bellingshausen Sea depending on the bathymetry. The frontal jet is closest to the shelf break in areas with a comparatively steep slope, such as between 90–92°W and 72–81°W. In contrast, the frontal jet is located further away from the shelf break in areas with a relatively moderate slope, such as between 82°W and 87°W. The frontal jet coincides with the 1.5 °C isotherm, separating warmer off‐shelf waters from colder waters further south, as shown by the long‐term mean of vertically averaged potential temperature (Figure 2a).

**Figure 4:**
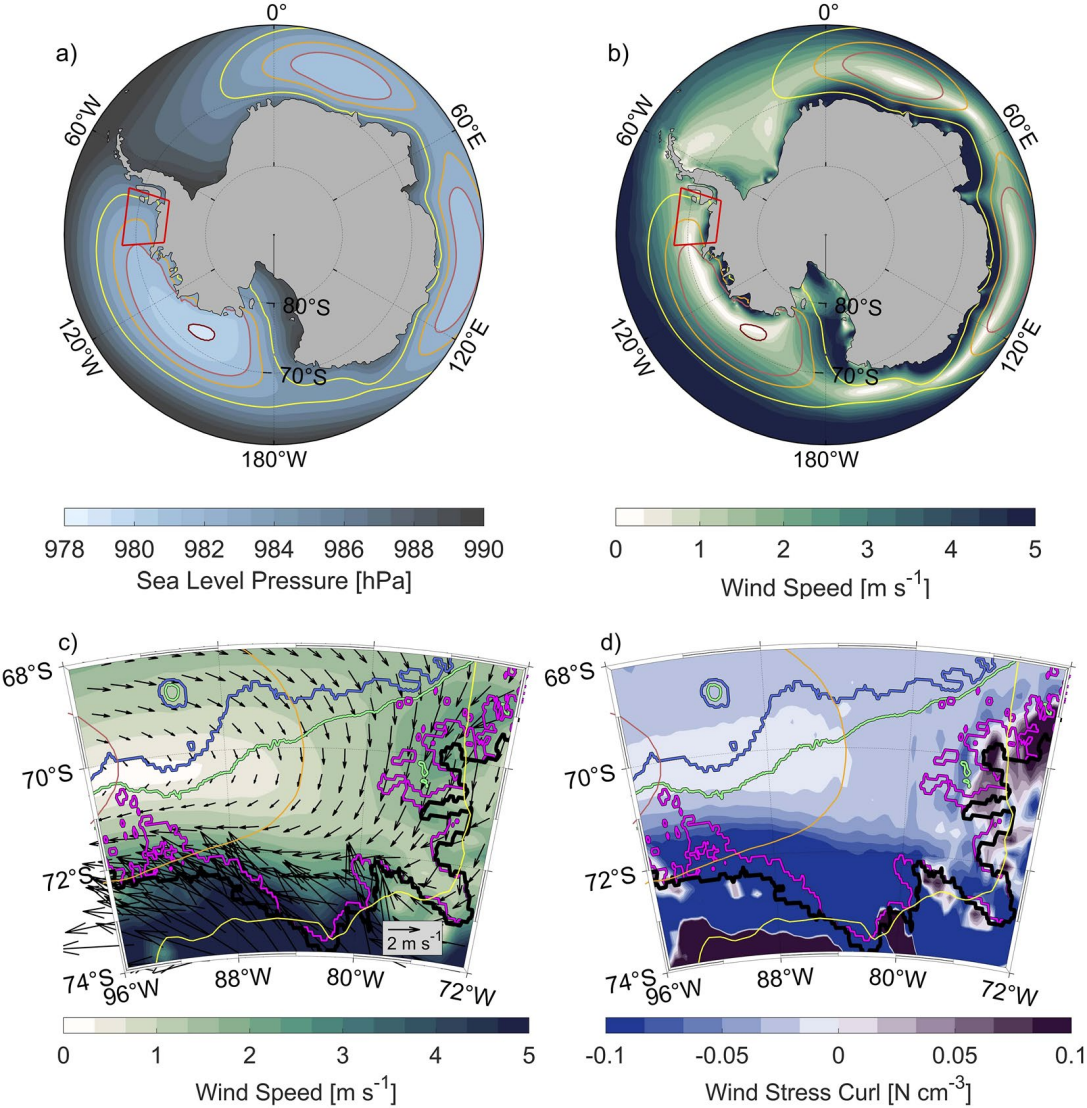
Long‐term mean from 1993 to 2018, extracted from ERA5, of (a) sea level pressure and (b) wind speed around Antarctica, (c) wind speed superimposed with arrows of wind velocity and (d) wind stress curl in the Bellingshausen Sea region. In all panels, the coastline of Antarctica is shown as a black contour, and sea level pressure contours are shown as thin green (980 hPa), red (982 hPa), orange (983 hPa), and yellow (985 hPa) lines. The low‐pressure region between 70°W and 180°W is known as the Amundsen Sea Low (ASL). For (c) and (d) isobaths are shown as in Figure 1 with exception of the 500‐m isobath.

Wind fields extracted from ERA5 (Figures 4b and 4c) reveal a cyclonic rotation around a low‐pressure system, the ASL (Figures 4a and 4b). The cyclonic rotation is also associated with a negative wind stress curl (Figure 4d). Large negative values of the wind stress curl occur south of 72°S and indicate Ekman upwelling which results in an uplift of isopycnals in this region. The zero wind stress curl at 90°–96°W above the continental slope coincides with the lowest sea level pressure and weakest wind intensity in the Bellingshausen Sea area and suggests that the ASL is centered further to the west (Figure 4a). Ekman transports modulated by wind direction and intensity are directed away from the central continental shelf, where a minimum in SSH supports the cyclonic gyre within 79°–89°W and 71°–74°S (Figure 2b). Assmann et al. (2005), using coupled ice‐ocean simulations, also found the region to be dominated by a cyclonic gyre with a similar longitudinal extent as in GLORYS. Moreover, recent studies observed individual cyclonic circulation features within the major troughs of the Bellingshausen Sea with inflow of mCDW along the eastern boundaries of the troughs up to the ice shelves (Schulze‐Chretien et al., 2021; Zhang et al., 2016). The on‐shelf transport appears to follow the 500 m contour in GLORYS; however, the cyclonic circulation within individual troughs on the Bellingshausen Sea continental shelf is not indicated clearly as the trough pathways are not well represented. Note that the troughs represented in GLORYS are narrower than the available bathymetric data products (e.g., R‐Topo2; Schaffer et al., 2016). Regions in the GLORYS troughs that are shallower than the actual depth may act as a topographic barrier by constraining the southward heat transport in lower layers associated with mCDW. The southernmost areas between 79°W and 88°W influenced by the cyclonic wind circulation exhibit strong meridional SSH gradients (Figure 2b) and reveal the Antarctic Coastal Current, which is most clearly identifiable in the vertically averaged current speeds from the surface to 300 m (Figure 2c). The Antarctic Coastal Current has also been identified in previous studies and reaches from the WAP through the Bellingshausen Sea into the Amundsen Sea (Assmann et al., 2005; Holland et al., 2010; Schubert et al., 2021).

Current speeds below 300 m (Figure 2d) are much weaker on the Bellingshausen Sea shelf than in the frontal jet. Velocities below 300 m near the shelf break suggest an inflow of warmer waters onto the shelf at 70.5°S and 89°–90°W. Once on the shelf, warm water masses flow roughly parallel to the shelf break, flooding the central and eastern shelf between 70°S and 71°S until they recirculate with the frontal jet at 76°W. Warmer temperatures on the shelf north of 71°S (Figure 2a) further indicate the inflow of warmer water (>1 °C) suggesting access of mCDW to the continental shelf. Summer sea ice concentrations (Figure 2e) throughout the central and eastern Bellingshausen Sea shelf vary between 50% and 70%. Winter sea ice concentrations (Figure 2f) are above 90% throughout the Bellingshausen Sea continental shelf with the exception of the southern continental shelf between 79°W and 87°W. Low sea ice concentrations in the southernmost region on the Bellingshausen Sea continental shelf indicate a coastal polynya near the Venable and Stange ice shelves. Strong northwestward winds south of 72°S (Figure 2c) might facilitate the transport of sea ice toward the western Bellingshausen Sea shelf, where the highest concentrations, between 80% and 90%, are found (Figure 2e) in summer. The long‐term summer and winter sea ice concentrations are represented well in GLORYS and reflect key features and patterns similar to satellite observations of summer and winter sea ice concentrations demonstrated by Parkinson and Cavalieri (2012).

A significant limitation of GLORYS is the lack of ice shelf cavities and thus a representation of water mass transformation as mCDW circulates within these cavities and becomes more buoyant due to the addition of glacial meltwater. This process plays an important role in the heat budget of the continental shelf (e.g., Couto et al., 2017), and its absence in these simulations may result in a positive bias (more ocean heat loss) in the surface heat loss discussed in Section 4. It has also been proposed that the ice shelf pump mechanisms contribute to setting the overturning magnitude in the Bellingshausen Sea (Ruan et al., 2021; Thompson et al., 2020) although the trough circulations that deliver warm water to the ice shelf cavities are largely barotropic features (Wåhlin et al., 2020), which should be adequately captured, to the extent that is possible with the model's bathymetry.

In summary, while the circulation on the shelf does not exactly match the observations, due to limitations in the model bathymetry and lack of ice shelf representation, we do find access of warmer water onto the Bellingshausen Sea continental shelf in the reanalysis long‐term mean. In the following section, we investigate the dominant patterns of spatial and temporal variability in water temperature below 300 m on the continental shelf.

## 3 Modes of Variability of Ocean Temperature on the Continental Shelf

For our analysis, Empirical Orthogonal Functions (EOF) are calculated from the vertically averaged potential temperatures below 300 m. Waters at these depths are most likely to enter ice shelf cavities and contribute to the melting process. To ensure that only deep water masses on the continental shelf, such as mCDW are considered, areas shallower than 300 m and deeper than 1,000 m are excluded from the calculation. In order to focus on interannual variability and to avoid seasonal effects in our study, we calculate EOFs from annual means.

**Figure 5:**
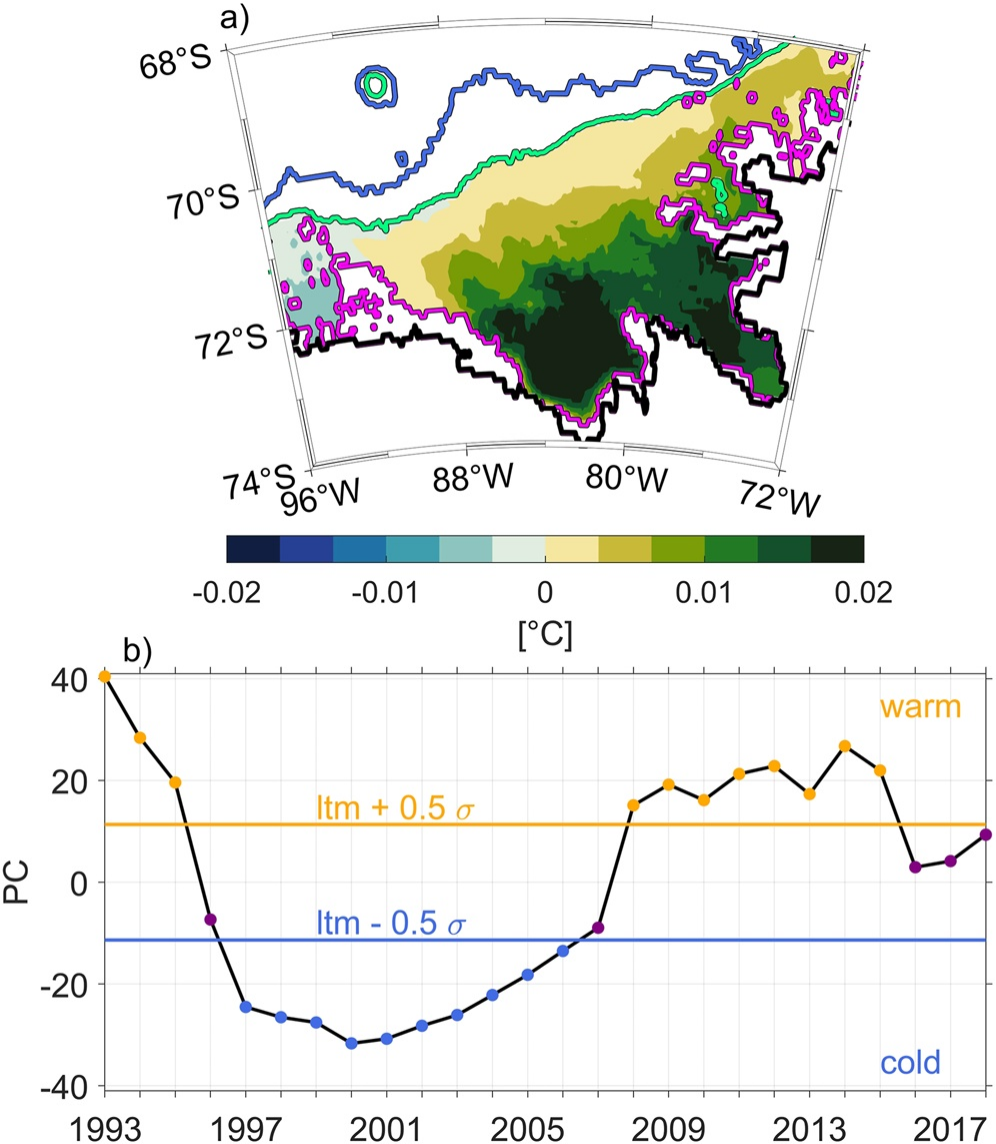
First Empirical Orthogonal Function (EOF) mode for potential temperature below 300 m on the Bellingshausen Sea continental shelf. The EOF has been calculated from 1993 to 2018 annual means, where areas deeper than 1,000 m and shallower than 300 m have been excluded. Isobaths are colored as in Figure 4. (b) Principal component (PC; unitless) of the first EOF mode. Horizontal lines represent upper and lower boundaries for warm (orange) and cold (blue) years which are calculated as ltm ±0.5*σ*, where ltm is the long‐term mean and *σ* is the standard deviation of the PC.

**Figure 6:**
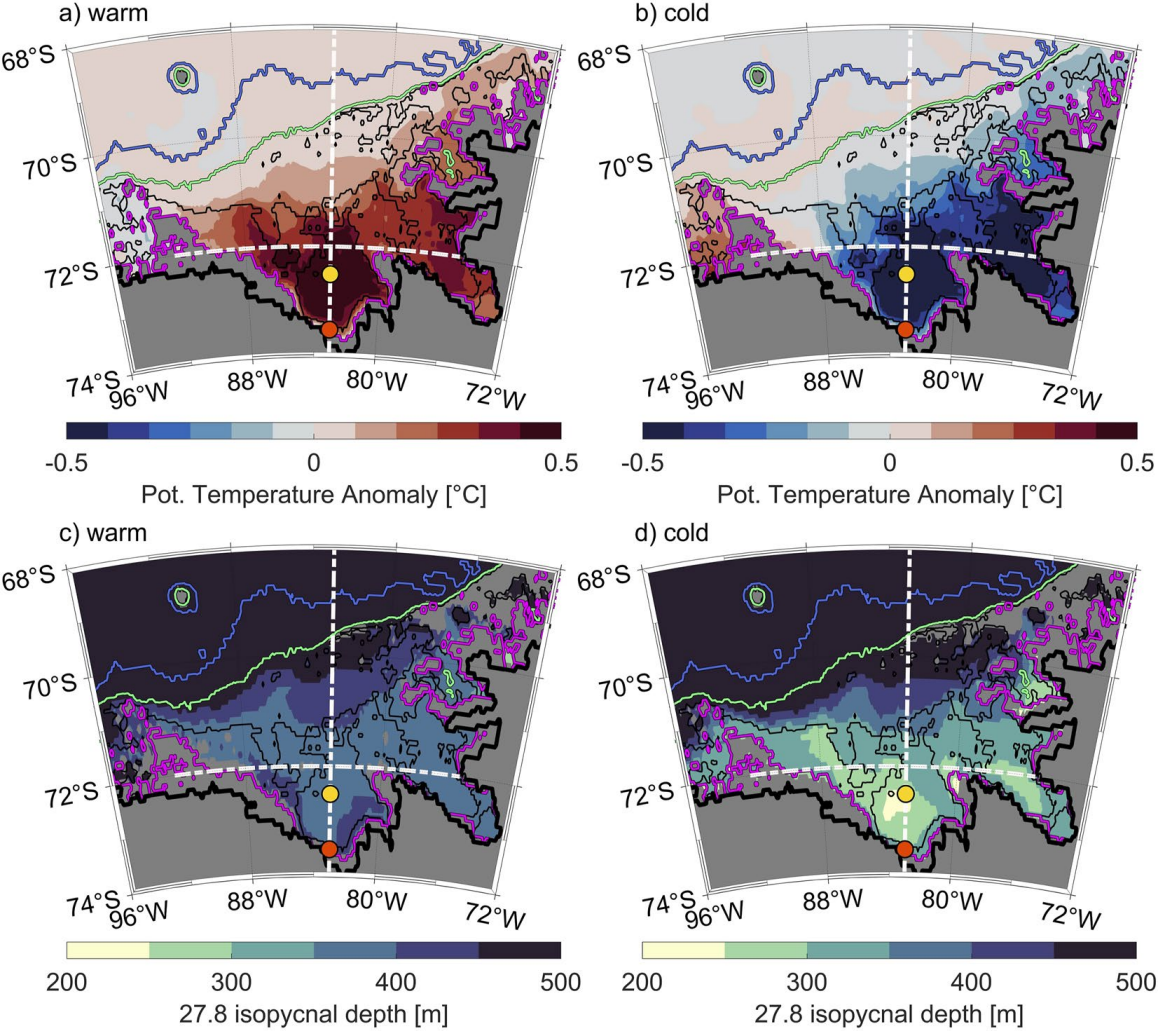
(a and b) Anomalies of vertically averaged potential temperature from 300 m to the bottom of the shelf, where positive anomalies (red) imply higher temperatures, and (c and d) composites of the 27.8 kg m^−3^ isopycnal depth, for the warm (a, c) and cold (b, d) regimes. Yellow and orange dots mark the locations where temperature and salinity profiles are taken to describe the vertical structure of water masses (Figures 7 and 14, respectively). White dashed lines show the meridional and zonal transect locations (shown in Figures 8 and 9). Isobaths are colored as in Figure 1.

This study focuses on the first EOF mode (Figure 5a), which describes the pattern of the most dominant mode of variability and explains 65% of the variance. Further modes explain <20% each and are not considered further. Large values (positive or negative) imply that a grid point has a large amplitude of temporal variability associated with this spatial pattern. The first EOF mode presents a weak amplitude of temporal variability at the shelf break throughout the central and eastern Bellingshausen Sea. This is because the temperature of the water below 300 m near the shelf break does not vary as much as that further south in Latady and Eltanin Bay (Figures 6a and 6b). South of 71°S EOF values increase toward coastal regions, where EOF maxima are predominantly found within Eltanin and Latady Bays near the coast. The western edge of the Bellingshausen Sea (west of 91°W) presents an EOF temperature variability that is out of phase compared with other parts of the shelf. This is likely due to the relatively shallow region between 90°W and 92°W, that may be considered the boundary between the Bellingshausen Sea and the Amundsen Sea, and thus between two different dynamical regimes.

The EOF time series (Figure 5b), hereinafter called principal component (PC), describes the weighted amplitude representing the spatial variability of temperature below 300 m and highlights the two main regimes (warm and cold). Using a weighted amplitude of variability is important for the purpose of defining warm and cold regimes, as the temperatures below 300 m on the Bellingshausen do not vary uniformly for all areas of the continental shelf (Figure 5a). Years in which the PC > ltm + 0.5*σ*, where *σ* is the standard deviation of the PC and ltm is the long‐term mean, are considered warm years (11 years in total, 1993–1995 and 2008–2015). Similarly, years in which the PC < ltm − 0.5*σ* are considered to be cold years (10 years in total, 1997–2006). The years 1993 and 2000 are the warmest and coldest years of the period, respectively. The remaining years that do not qualify as either warm or cold will be referred to as transition years. Periods of pronounced warm and cold years mostly agree with those identified by Nakayama et al. (2018), who described simulated colder and warmer mCDW in the years from 2001–2006 to 2009–2014 using a regional configuration of the Massachusetts Institute of Technology general circulation model (MITgcm) for the Amundsen Sea and Bellingshausen Sea regions. There exist slight differences in the timing of warm and cold years in GLORYS and the work of Nakayama et al. (2018). A possible explanation for these differences is that the ECCO‐v4 surface forcing was used in the MITgcm simulation, which is based on ERA‐Interim but differs slightly (Nakayama et al., 2017). The separation of warm and cold years in the neighboring Amundsen Sea near the Dotson ice shelf by Jenkins et al. (2018) only partly agrees with our determinations of warm and cold periods in the Bellingshausen Sea. Oceanic regime transitions derived from hydrographic observations discussed by Jenkins et al. (2018) indicated a cold period from 2000 to 2003, warm period between 2004 and 2011, and a further cold period from 2012 to 2016, where 2009 was the peak warm phase and 2006–2007 intermediate years. A clear difference between the Bellingshausen and Amundsen Seas with respect to warm and cold regimes occurs during the most recent cold period from 2012 to 2016 in the Amundsen Sea. The Amundsen Sea cold period has been described extensively in previous studies using observations (Jenkins et al., 2018; Webber et al., 2017) and simulations (Dotto et al., 2019; Dutrieux et al., 2014). Dutrieux et al. (2014) and Webber et al. (2017) demonstrated that ocean conditions are partly attributable to atmospheric forcing and sea ice formation in the Amundsen Sea. In contrast, the Bellingshausen Sea EOF mode suggests 2012–2015 to be warm years and 2016–2018 to be transition years. This suggests that water temperatures in the Amundsen and Bellingshausen Seas are not always in phase and may be controlled by different processes. The findings agree with the out of phase EOF temperature variability at the western edge of the Bellingshausen Sea (west of 91°W, transition to the Amundsen Sea, Figure 5a).

We use a PC‐based definition of warm and cold years to calculate warm and cold composites (PC‐weighted mean of all warm and all cold years) and anomalies (composite minus the long‐term mean fields described in Section 2). The anomalies (Figures 6a and 6b) present anomalously warm and cold temperatures below 300 m consistent with the variability pattern provided by the first EOF mode and are, as expected, correlated to its PC (not shown). Generally, the spatial distribution indicates that on‐shelf temperatures south of 71°S are increasingly warm from the shelf break toward the coast for the warm regime and increasingly cold toward the coast for the cold regime, as expected from the EOF map where the first EOF mode increases toward the coast (Figure 5a). Maximum temperature anomalies of >0.5 °C are found within Eltanin and Latady Bays in the south of the Bellingshausen Sea continental shelf.

**Figure 7:**
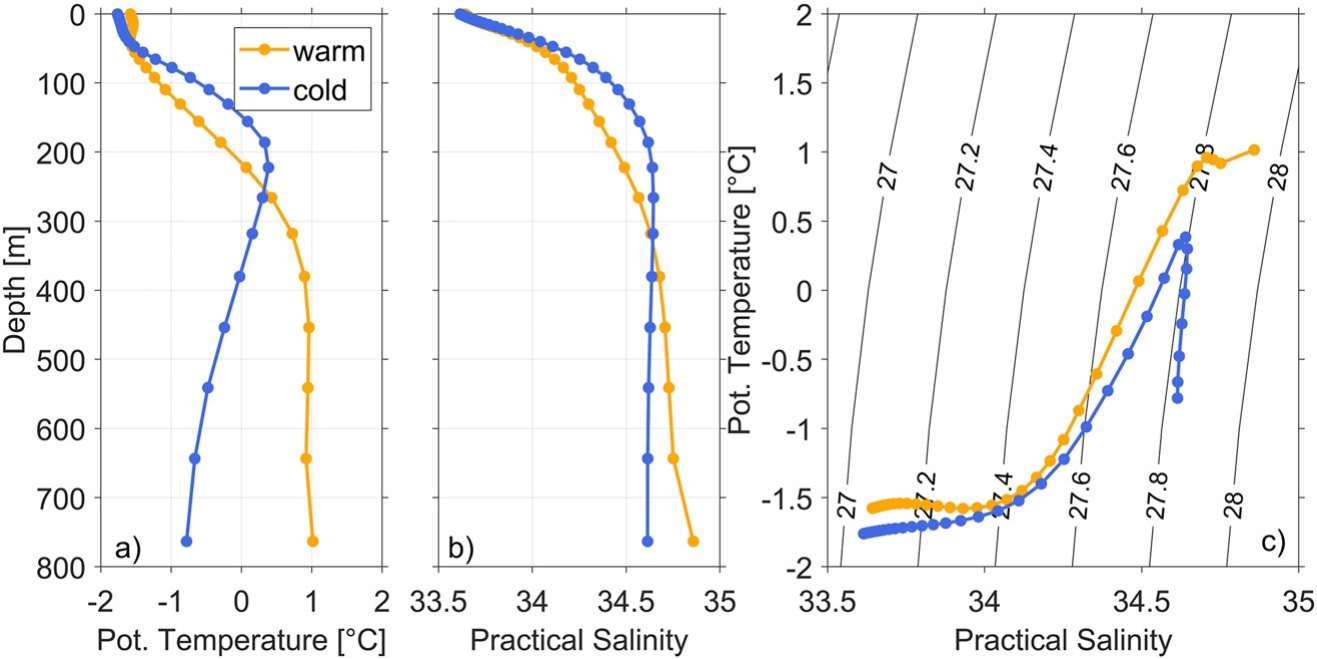
Vertical profiles of (a) potential temperature and (b) practical salinity along with (c) *θ*‐S diagrams for the warm (orange) and cold (blue) composites with contours of potential density. The profiles are taken from 72.5°S to 83°W (Figure 6, yellow dots), a location with high interannual variability in the temperature below 300 m.

To explore the presence of mCDW and the difference in water mass stratification on the Bellingshausen Sea continental shelf for warm and cold regimes, we select a vertical profile at a location (83°W and 72.5°S, Figure 6, yellow dots) characterized by the strongest anomalies in temperature below 300 m within Eltanin Bay (Figure 7). Below the relatively fresh surface mixed layer near the freezing point, identified as Antarctic Surface Water, the cold regime has a temperature maximum of 0.5 °C at a depth of about 250–300 m. This temperature maximum is colder than that of the warm regime, suggesting more modification of CDW in the cold regime. Water masses below the thermocline cool to about −0.7 °C and slightly freshen toward the seabed. In contrast, the warm regime presents a slightly warmer surface mixed layer and a warmer temperature maximum up to almost 1 °C, coinciding with increased salinity of up to 34.84. This water mass, identified as weakly modified CDW, extends throughout the remaining water column down to the seabed. These findings show the increased presence of mCDW in Eltanin Bay in the warm regime, with temperatures almost 2 °C greater than in the cold regime.

**Figure 8:**
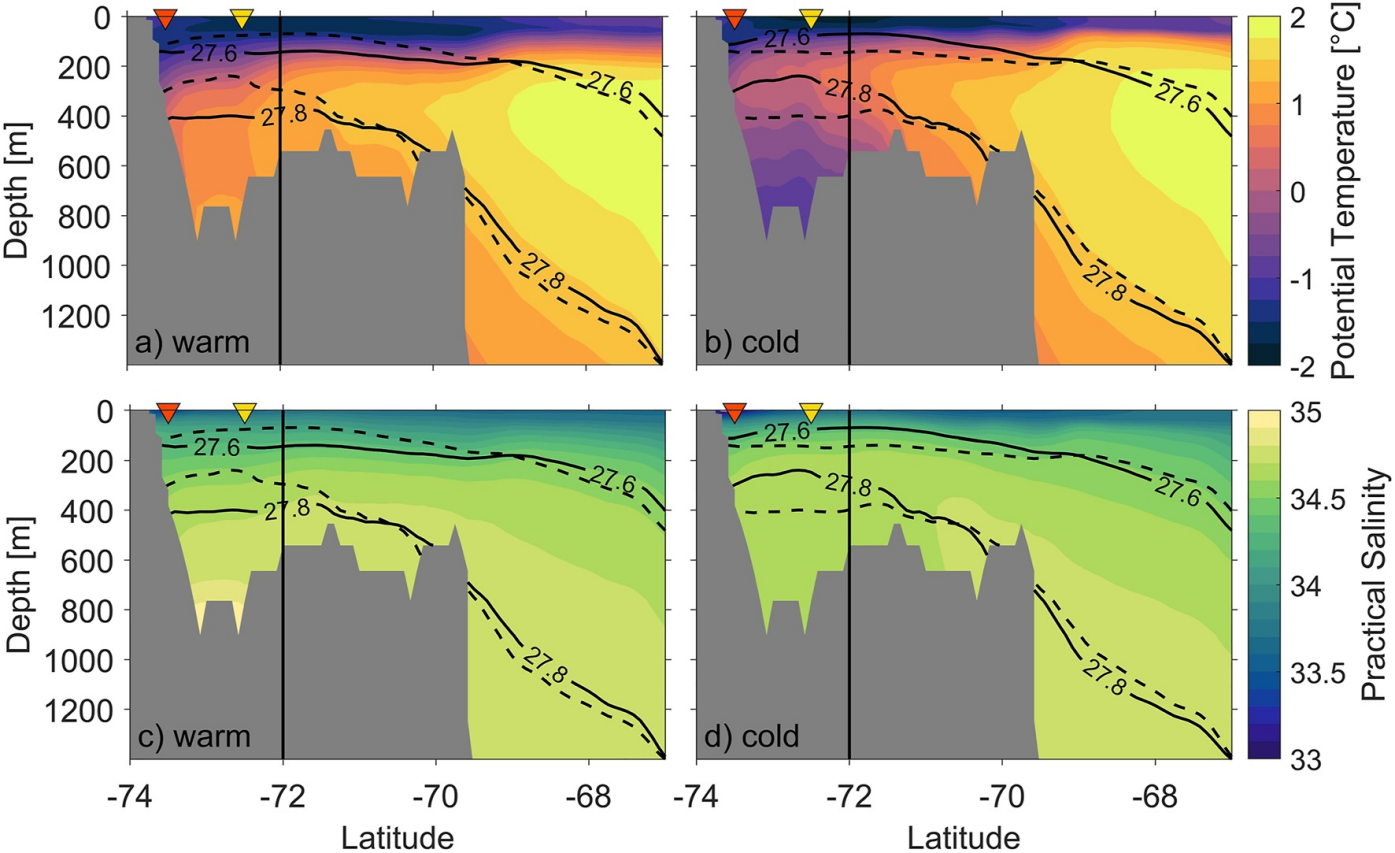
Composites of the meridional transect covering 67–74°S at 83°W (as displayed in Figure 6, white dashed lines) from GLORYS with (a, b) potential temperature and (c, d) practical salinity for the warm (a, c) and cold (b, d) regimes. Solid black lines on all panels are potential density for the warm (a, c) and cold (b, d) composites, whereas dashed black lines show the potential density of the opposite regime to highlight the differences between warm and cold regimes. The triangles indicate the locations of vertical profiles at 73.5°S (orange) and 72.5°S (yellow) as marked in Figure 6. The shaded areas (gray) display the Bellingshausen Sea shelf bathymetry of the meridional transect crossing the shelf break. The vertical black line shows the location at 83°W, 72°S, where the meridional and zonal transects intersect.

**Figure 9:**
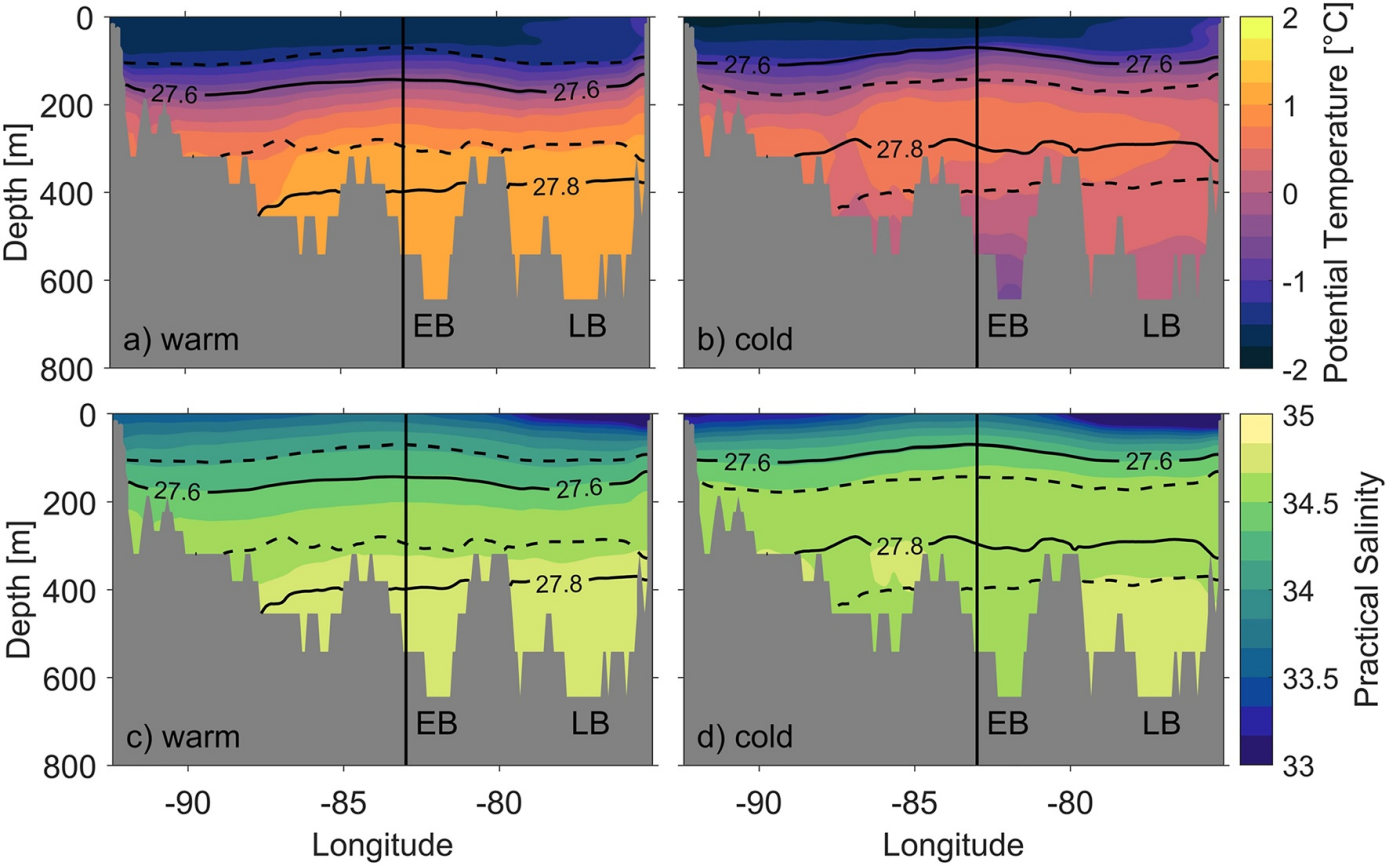
Composites of the zonal transect covering 75–92.5°W at 72°S (as displayed in Figure 6, white dashed lines) from GLORYS with (a, b) potential temperature and (c, d) practical salinity of the warm (a, c) and cold (b, d) regimes. Solid black lines on all panels are potential density for the warm (a, c) and cold (b, d) composites, whereas dashed black lines show the potential density of the opposite regime to highlight the differences between warm and cold regimes. The shaded areas (gray) display the Bellingshausen Sea shelf bathymetry of the zonal transect crossing the Bellingshausen Sea continental shelf. The acronyms EB and LB mark the locations of Eltanin and Latady Bays in the zonal transect. The vertical black line shows the location at 83°W, 72°S, where the meridional and zonal transects intersect.

To identify the vertical and spatial extent of mCDW, we show a meridional transect crossing the shelf break and a zonal transect crossing the continental shelf. The meridional and zonal transects intersect at 72°S as shown in Figure 6 (white dashed lines). Note that the zonal transect is terminated by land at the eastern end, and by very shallow water (<30 m) at the western end. The meridional and zonal transects (Figures 8 and 9) demonstrate that the lower water column is occupied by a colder and fresher water mass south of 71.3°S in the cold regime, whereas the lower water column in the warm regime is dominated by mCDW. We define two isopycnals (27.6 and 27.8 kg m^−3^, potential density) to identify upper and lower boundaries for mCDW on the continental shelf. The 27.6 and 27.8 kg m^−3^ isopycnals at the shelf break are in a similar depth in the cold and the warm regimes (Figure 8), which suggests that neither the warm nor the cold regime would allow greater access of mCDW onto the continental shelf in the meridional transect. Composites of the 27.8 kg m^−3^ isopycnal depth (Figures 6c and 6d) confirm the same isopycnal depth above shelf break and continental slope for both regimes. Both isopycnals are shallower in the central and southern Bellingshausen Sea in the cold regime (Figures 8b, 8d and 9b, 9d) than in the warm regime (Figures 8a, 8c and 9a, 9c). Specifically, the 27.8 kg m^−3^ isopycnal in the cold regime indicates that mCDW does not occupy deeper layers of the water column within Eltanin Bay (Figures 8b and 8d). Composites of the 27.8 kg m^−3^ isopycnal depths (Figures 6c and 6d), confirm the stronger uplift of this isopycnal in the cold regime, predominantly within Eltanin and Latady Bays and near coastal regions. A possible explanation for the shallower isopycnals in the central and southern Bellingshausen Sea in the cold regime is the heaving of isopycnals in response to changes in SSH. The SSH levels for both regimes are discussed in Section 4 in more detail.

In order to establish whether water mass properties found in GLORYS are representative of conditions in the Bellingshausen and Amundsen Seas, we compare the model output with existing hydrographic observations from these shelf seas. Previously observed hydrographic transects crossing the Belgica and Latady Troughs in 2007 and 2018/19 (Ruan et al., 2021; Schulze‐Chretien et al., 2021) show similar characteristics to GLORYS for the zonal transect in the warm regime (Figure 9a), where mCDW occupies the lower layers of the water column from 300 m to the seabed with similar water mass properties and maximum temperatures of up to 1.6 °C. Jenkins et al. (2018), on the other hand, observed the water mass stratification in a cold year near Dotson ice shelf in the Amundsen Sea, which indicated that mCDW is only present in lower layers of the water column reaching from 700 to almost 1,000 m. These findings are substantially different to the water mass stratification of cold years presented by GLORYS in the Bellingshausen Sea, where both transects (meridional and zonal) show mCDW higher up in the water column with uplifted 27.6 and 27.8 kg m^−3^ isopycnals in the southern and central Bellingshausen Sea in the cold regime. The zonal transect from GLORYS of the warm regime (Figure 9a) shows a similar water mass stratification in the south of the Bellingshausen Sea as compared to the observed water mass stratification of Jenkins et al. (2018) in a warm year. The meridional and zonal transects of the warm regime demonstrate the presence of mCDW on the Bellingshausen Sea continental shelf including in Eltanin and Latady Bays, and in close proximity to the coastline and thus theoretically close to the ice shelf cavities.

We have shown that the water mass stratification differs significantly between the warm and the cold regimes in GLORYS on the southern Bellingshausen Sea continental shelf. Meridional and zonal transects and vertical profiles indicate that differences mainly occur in the lower layers of the water column and are related to the presence of mCDW in Eltanin and Latady Bays.

## 4 Mechanisms Leading to Warm and Cold Regimes on the Continental Shelf

We now seek to determine whether net meridional heat transport *Q*_*hf*_ or net air‐sea flux *Q*_*surf*_, dominate the temporal change in heat content $\frac{d\bar{H}}{dt}$ south of 72°S.

The monthly mean heat content for the volume south of 72°S is defined as

(1) $$\bar{H}=\int_{x_{w}}^{x_{e}}\int_{y_{s}}^{y_{n}}\int_{-h}^{0}\rho c_{p}\left(\bar{\theta }-\theta_{\mathrm{ref}.}\right)dxdydz,$$

where $\bar{}$ indicates the monthly average, *ρ* is the potential density, *c*_*p*_ = 3,982 J (kg K)^−1^ is the specific heat capacity, and *θ* is the potential temperature. *θ*_*ref*_ = −1.8 °C is a reference temperature, which for simplicity we take as the coldest temperature recorded in GLORYS in this domain, *x* is the zonal distance where *x*_*w*_ and *x*_*e*_ define the zonal limits, *y* is the meridional distance with *y*_*s*_ (latitude of the coast) and *y*_*n*_ (72°S) defining the meridional limits, *z* is height, and *h* is the local seabed depth of GLORYS.

We calculate the monthly mean meridional heat flux $\bar{F_{h}}$ through each grid cell of the zonal transect at 72°S as

(2) $${\bar{F}}_{h}=\rho c_{p}\bar{v}\left(\bar{\theta }-\theta_{\text{ref}}\right),$$

where $\bar{v}$ is the meridional velocity component normal to the transect, positive northwards.

Then the monthly mean net heat transport $\bar{Q_{hf}}$ through the zonal transect (as indicated in Figure 6, white dashed lines) is given by

(3) $$\bar{Q_{hf}}=\int_{x_{w}}^{x_{e}}\int_{-h}^{0}\bar{F_{h}}dxdz.$$

Note that the net volume transport through the entire zonal transect is near zero on average (≈0.02 ± 0.02 Sv) and thus inflow equals outflow as the net evaporation minus precipitation, ice melt and river run‐off are negligible in this region in GLORYS.

The air‐sea‐ice flux $\bar{Q_{\mathrm{surf}}}$ within the area south of 72°S is not provided by the reanalysis output but is deduced as the difference between the two terms above

(4) $$\bar{Q_{\mathrm{surf}}}=\frac{d\bar{H}}{dt}-\bar{Q_{hf}},$$

where $\frac{d\bar{H}}{dt}$ is the change in heat content over the monthly time interval. Note that the air‐sea flux $\bar{Q_{\mathrm{surf}}}$ includes processes involved with sea ice formation and melt as well as the air‐sea heat fluxes. The annual‐mean net volume heat content, heat transport, and heat flux for a given year are then

(5) $$<\frac{dH}{dt},Q_{hf},Q_{\mathrm{surf}}>=\frac{\sum \frac{\bar{dH}}{dt},\bar{Q_{hf}},\bar{Q_{\mathrm{surf}}}}{12},$$

where <⋅> indicates the average of the monthly means within a given year.

**Figure 10:**
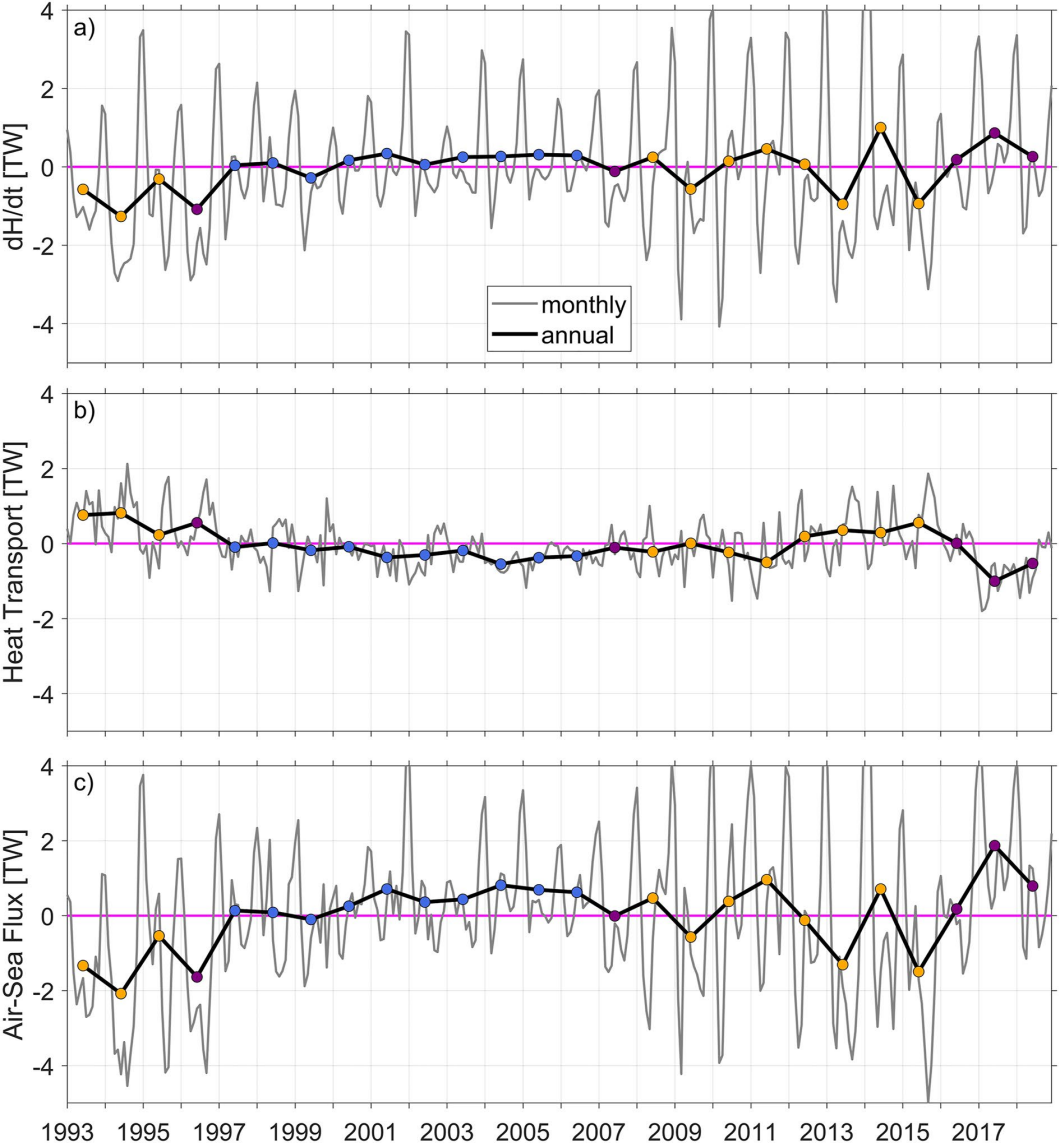
Monthly (gray lines) and annual means (black lines) of the net (a) temporal heat content change $\frac{dH}{dt}$, (b) heat transport *Q*_*hf*_ through the zonal transect at 72°S (positive northwards) and (c) air‐sea flux *Q*_*surf*_ (positive air to sea) for the area south of 72°S in the southern Bellingshausen Sea (see Equations (1), (2), (3), (4), (5) in Section 4 for further details). Note that the air‐sea flux *Q*_*surf*_ includes processes involving sea ice formation and melt and that annual mean values are positioned in the center of the averaged year. Colored dots represent cold (blue), transition (purple), and warm (orange) years (as in Figure 5e). The zero line is highlighted in magenta.

The time series of $\frac{dH}{dt}$, *Q*_*hf*_ and *Q*_*surf*_ from 1993 to 2018 are shown in Figure 10. The basic ocean heat budget illustrates the variability of $<\frac{dH}{dt}>$ south of 72°S associated with the variability of $<Q_{hf}>$ (crossing 72°S) and $<Q_{surf}>$ and further highlights the differences in the heat budget components for warm and cold years. The monthly means illustrate the seasonal variations, with positive $\frac{d\bar{H}}{dt}$ and $\bar{Q_{\mathrm{surf}}}$ (surface heat uptake) during summer and negative $\frac{d\bar{H}}{dt}$ and $\bar{Q_{\mathrm{surf}}}$ (surface heat loss) during winter (Figure 10). In the following, we assess annual means to determine which of the above mentioned processes are dominant in the warm and cold regimes. $<\frac{dH}{dt}>$ is negative during the warm regime from 1993 to 1996, also shown by the PC (Figure 5b). This decrease in heat content is driven by a net northward $<Q_{hf}>$ and negative $<Q_{surf}>$ during those years until the cold regime begins in 1997. After entering the cold regime, $<\frac{dH}{dt}>$ is slightly positive and remains almost constant until 2008. The gradual increase in temperature over time is driven by a net southward $<Q_{hf}>$ and a slightly positive $<Q_{surf}>$ (ocean heat uptake). This gradual warming over time results in a transition to the second warm regime in 2008. During the warm regime (2008–2015), $<\frac{dH}{dt}>$ is more variable, also seen in higher variability of monthly means of heat budget variables. During the second warm regime, $<Q_{hf}>$ is northward apart from 2008, 2010, and 2011. $<Q_{surf}>$ vary significantly during this period. A positive $<Q_{surf}>$ in 2014 results in a short‐term increase in $<\frac{dH}{dt}>$ even though $<Q_{hf}>$ is northward. In 2015, $<Q_{surf}>$ is negative, due to increased heat loss in winter and weaker heat uptake in summer. Thus, the change in heat content arises as a residual between a large values of heat uptake in the summer and heat loss in the winter. Overall, we find that water masses within Latady and Eltanin Bays experience warming (increasing heat content over time) during the cold regime and cooling (decreasing heat content over time) during the warm regime. The heat budget for the southern Bellingshausen Sea suggests that air‐sea fluxes dominate over lateral ocean heat transport, at least in the GLORYS. Note that the heat budget may be impacted by the lack of ice shelf cavities in GLORYS. Nonetheless, this finding is surprising as the southward heat transport associated with mCDW is often thought to be the main driver of enhanced ice shelf melt on the West Antarctic continental shelves.

We have shown that warm and weakly modified CDW can reach Eltanin and Latady Bays in the south of the Bellingshausen Sea continental shelf in GLORYS. Furthermore, we have demonstrated that a net southward heat transport and warming of the shelf occur primarily in cold years, which indicates a warming process within the cold regime. Conversely, the warm regime experiences a cooling of the waters in Eltanin and Latady Bays. Therefore, we now seek to understand which dynamical processes and mechanisms are involved in controlling the change in heat content south of 72°S of subsurface waters within Eltanin and Latady Bays.

**Figure 11:**
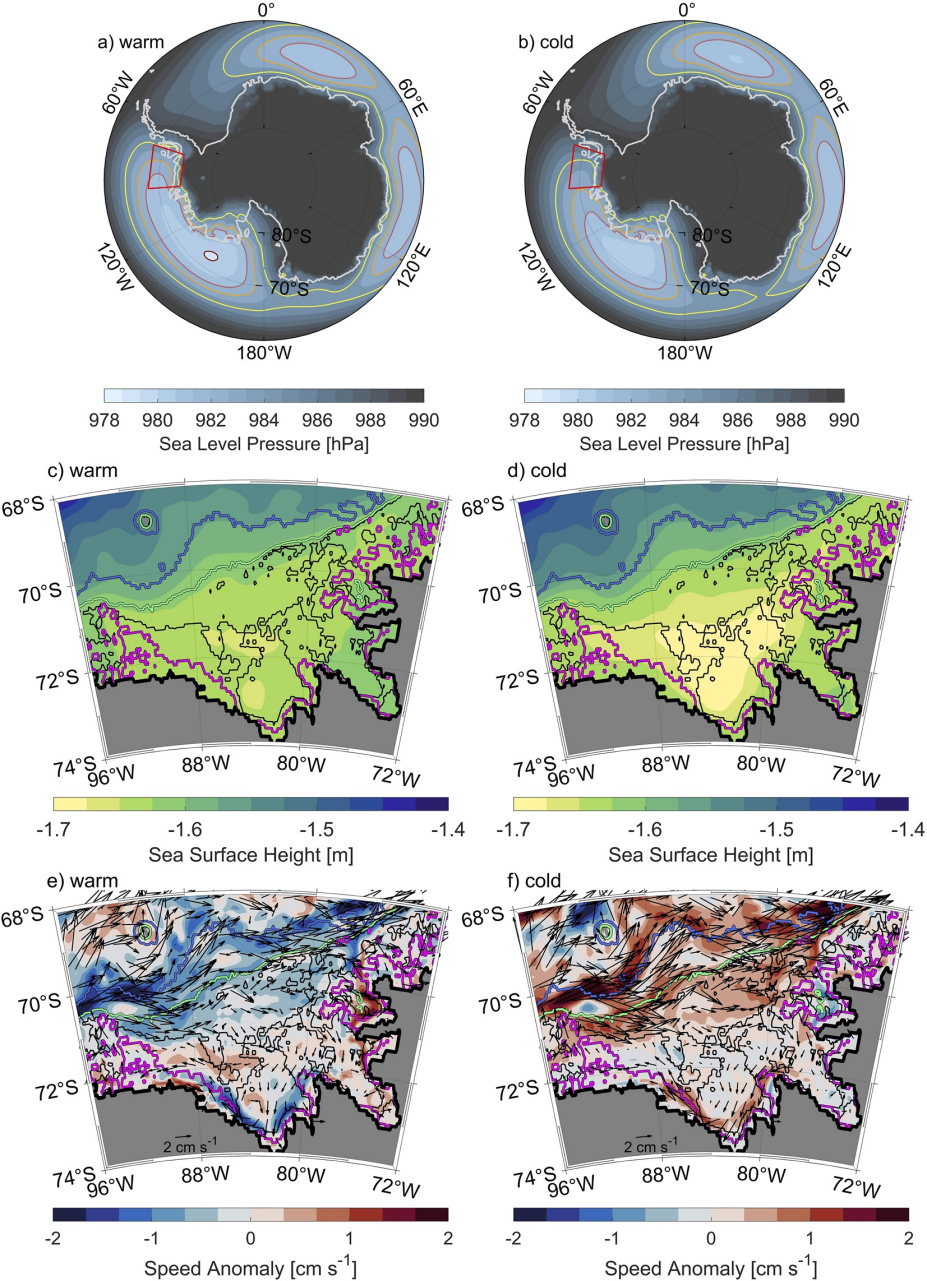
Composites of (a, b) sea level pressure and (c, d) sea surface height (SSH) for the warm (a, c) and cold (b, d) regimes. Anomalies of current speeds averaged from the surface to 300 m with superimposed current velocities from composites of the warm (e) and cold (f) regimes. The red box (a, b) highlights the Bellingshausen Sea region and sea level pressure contours and isobaths are colored as in Figure 1. Positive anomalies (e and f, red) imply higher current speeds.

In Section 1, we hypothesized that the inflow of mCDW into Eltanin and Latady Bays is associated with changes in the strength and/or an intensification of the ASL. The long‐term mean of sea level pressure shows that the ASL extends into the Bellingshausen Sea (Figure 4). Composites of the sea level pressure for the warm and cold regimes (Figures 11a and 11b) indicate a change in both the ASL's intensity and its eastern extent into the Bellingshausen Sea. The results show that in the cold regime the ASL is weaker and does not extend as far east into the Bellingshausen Sea compared with the warm regime, where the ASL is stronger and extends further east into the Bellingshausen Sea.

**Figure 12:**
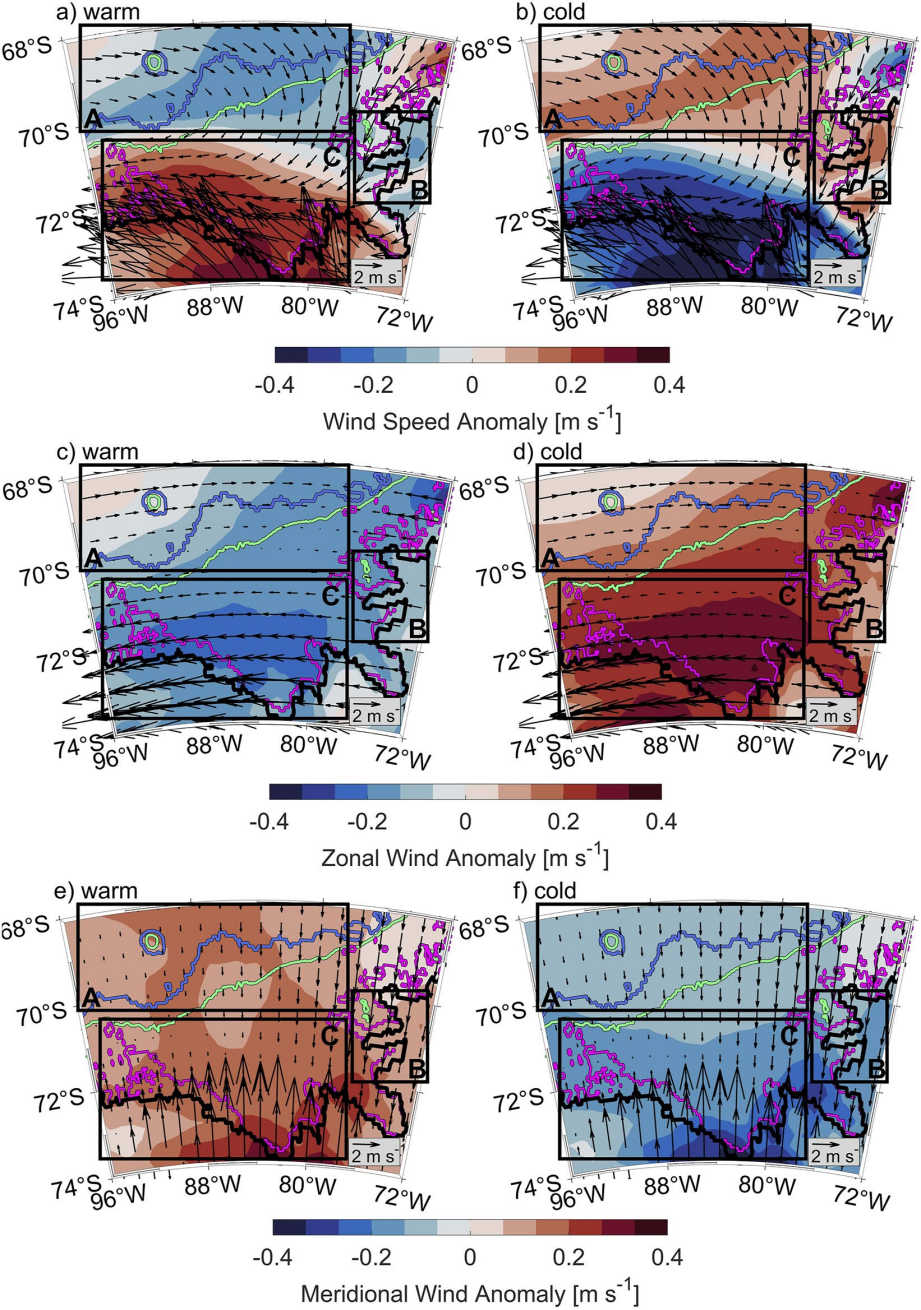
Anomalies of (a, b) wind speeds, (c, d) zonal wind components, and (e, f) meridional wind components. All subfigures (a)–(f) are superimposed with velocity vectors from the composites for the warm (a, c, e) and cold (b, d, f) regimes. Boxes A, B, and C highlight areas discussed in the text. Isobaths are colored as in Figure 4.

To understand the changes in atmospheric circulation between the warm and the cold regimes, we consider the zonal and meridional wind components separately. Along with meridional and zonal wind vectors, we consider the magnitude of wind speeds from both components compared with the long‐term mean (Figure 12). Note that positive anomalies are directed eastward for zonal winds and so represent either an increase in eastward or a decrease in westward wind speed. Similarly, positive anomalies for the meridional component are directed northward and so represent an increased northward wind speed or decreased southward wind speed.

For the purposes of the following discussion, we divide the study region into three boxes, as shown in Figure 12. Box A covers the area offshore, and over the continental slope and shelf break, where Ekman transport away from the continental shelf occurs. Box B covers the area of the study region which is bounded by land to the east. Box C covers the southernmost areas, including Eltanin Bay and the coastal polynya mentioned in Section 2. Since the Eltanin and Latady Bays show a similar overall pattern of temperature variability (Figure 5a), Latady Bay is not included in Box C. We confine our analysis to Eltanin Bay and the coastal polynya region, which are representative of the wider southern Bellingshausen Sea shelf.

Box A, which covers the shelf break region, shows a time‐mean wind direction to the southeast and is investigated to discuss the Ekman transport away from the Bellingshausen Sea continental shelf in the warm and cold regimes. The wind direction and intensity show significant seasonality in Box A (Figure S2 in Supporting Information S1). In summer, the ASL is located further east and further north, leading to westward winds in Box A. In winter, the ASL is located further west and further south, leading to eastward winds in Box A. The long‐term mean winds are similar to the winter winds indicating that the wind patterns displayed for winter are representative of much of the year, so anomalies in wind for the cold and warm regimes shown for winter will dominate over those for summer. In winter (and in the long‐term mean), the cold regime is characterized by greater wind speeds, with an increase in both eastward and southward wind components (Figure S3 in Supporting Information S1 and Figures 12b, 12d, and 12f). This means that the Ekman transport away from the continental shelf increases and leads to lower SSH on the shelf (Figure 11d). The summer wind speeds show an increase in westward and northward winds (in the west of Box A) and southward winds (in the east of Box A) in the cold regime compared with the summer long‐term mean. This will increase Ekman transport onto the continental shelf in the summer, but as noted previously this will be a smaller effect than the increased Ekman transport off the continental shelf in the rest of the year. The net effect over the whole year will be to increase SSH gradients over the continental slope, consistent with greater current speeds seen in the frontal jet (Figure 11f). In the warm regime, wind speeds in winter (and in the long‐term mean) and both the eastward and southward wind components (Figure S4 in Supporting Information S1 and Figures 12a, 12c, and 12e) weaken, so the Ekman transport (while still directed away from the shelf) reduces. Resulting SSH gradients over the continental slope (Figure 11c) reduce, consistent with lower speeds in the frontal jet (Figure 11e). The proximity of the frontal jet to the shelf break (Figures 11e and 11f) can be seen as an indicator for the proximity of CDW to the shelf break. Our results do not indicate a change in the proximity of the frontal jet to the shelf break in the warm or cold regimes. Thus, the proximity of CDW to the shelf break remains unchanged, but the increased current speeds in the cold regime are consistent with the net southward heat transport in cold years. The unchanged proximity of CDW to the shelf break is also confirmed by composites of potential temperature and practical salinity of warm and cold regimes (not shown), which do not show significant differences in the shelf break area. Overall, Box A displays increased Ekman transport off the shelf, increased SSH gradients over the continental slope and increased frontal jet intensity in the cold regime.

Box B, which covers the eastern Bellingshausen Sea, shows a time‐mean wind direction to the south and is investigated to discuss the potential impacts of wind direction and intensity on SSH and potential impacts on the Antarctic Coastal Current in warm and cold regimes. This region shows only minor differences between winter and summer (Figures S2, S3, and S4 in Supporting Information S1), so the seasonality is not discussed further. The cold regime shows increased southward winds and slightly weakened westward winds (Figures 12d and 12f), so overall wind speeds (Figure 12b) increase. The increased southward winds in the cold regime increase the Ekman transport toward the eastern boundary of the Bellingshausen Sea and possibly southeast into Latady Bay. These conditions likely explain why the overall decrease in SSH on the continental shelf in the cold regime is more pronounced in the center of the Bellingshausen Sea and Eltanin Bay, and less pronounced toward the eastern boundary and within Latady Bay (Figure 11d). The net result is that the zonal SSH gradients increase in Box B in the cold regime, consistent with the increase in the south‐westward flowing Antarctic Coastal Current in this area (Figure 11f). Conversely, the warm regime demonstrates decreased wind speeds in Box B (Figure 12a), reducing the Ekman transport and thus the SSH gradients and the strength of the Antarctic Coastal Current (Figure 11e).

**Figure 13:**
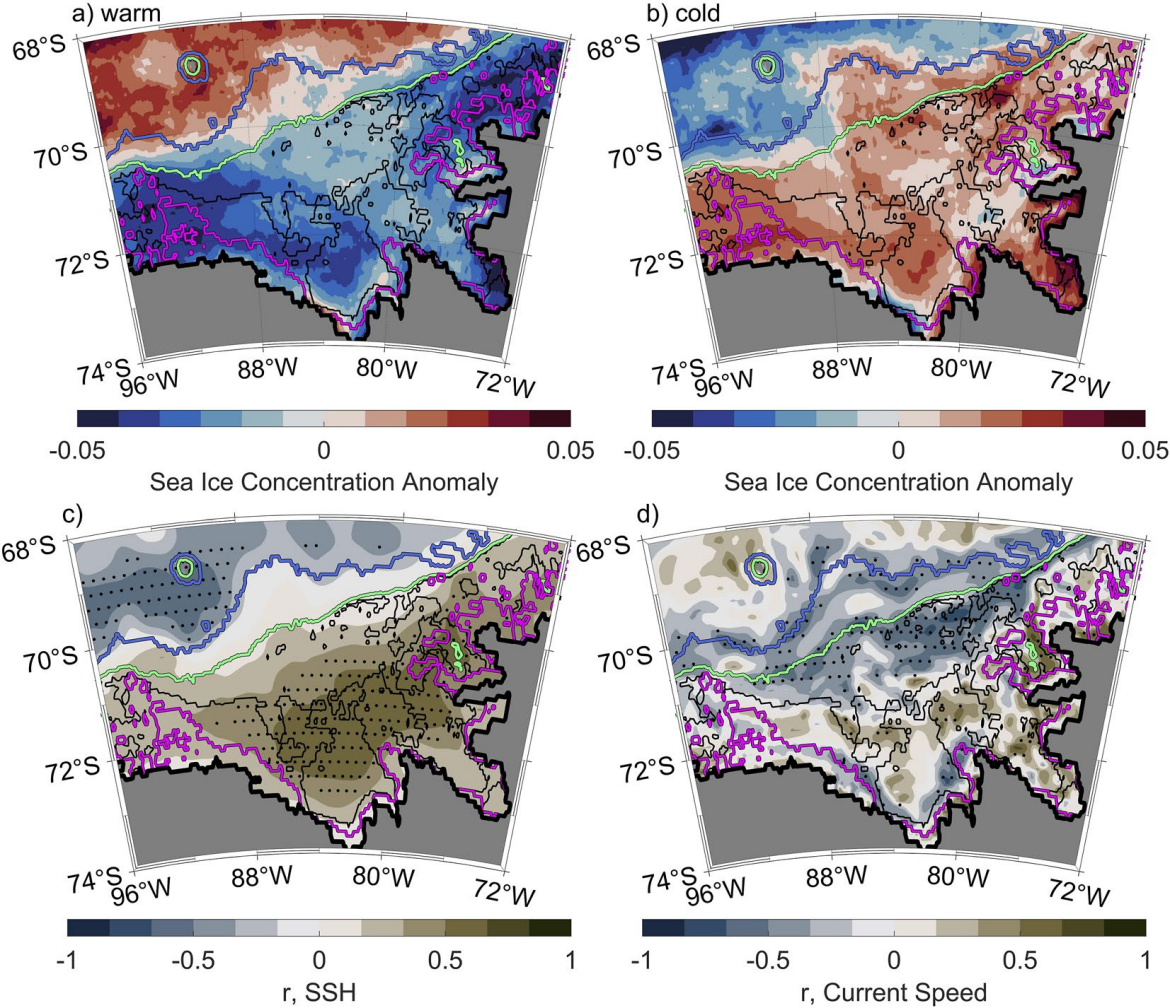
Sea ice concentration anomalies for the warm (a) and cold (b) composites. Map of the correlation between the principal component (PC) of the Empirical Orthogonal Functions (EOF) for (c) sea surface height (SSH) and (d) current speed averaged from the surface to 300 m. Stippling on (c) and (d) indicates statistically significant areas at a confidence level of 95%, with the critical values estimated by bootstrapping. Isobaths on panels (a)–(d) are colored as in Figure 1.

**Figure 14:**
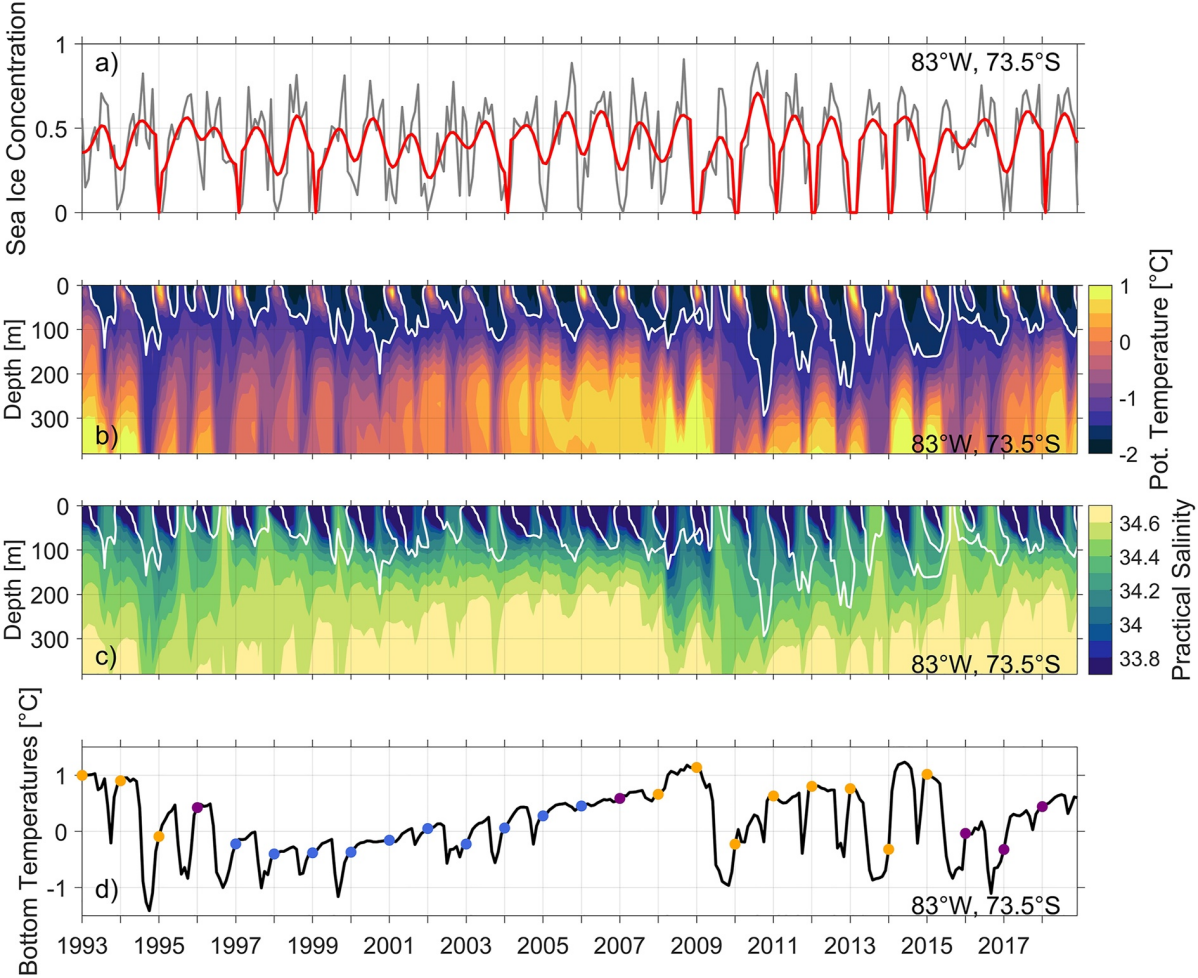
(a) Time series at 83°W, 73.5°S, showing: (a) monthly sea ice concentration (gray) and monthly sea ice concentration smoothed over 12 months (red), Hovmöller diagrams of (b) potential temperature and (c) practical salinity and (d) bottom temperatures. The white contour marks the −1.5 °C isotherm associated with cold, dense water formation. The colored dots in (d) indicate bottom temperatures for the years of the warm and cold regimes as defined in Figure 5.

Box C, which covers the southern Bellingshausen Sea, shows a time‐mean wind direction to the northwest and is investigated to highlight impacts of wind direction and intensity on sea ice concentration, heat loss to the atmosphere and cold, dense water formation in warm and cold regimes. In Box C, it is the warm regime that shows increased wind speeds (Figure 12a), with an increase in both the westward and northward wind components (Figures 12c and 12e) especially in winter. We suggest that these wind conditions are responsible for reduced sea ice concentration in the warm regime (Figure 13a), where sea ice is more rapidly blown away from the coast to the northwest, which enlarges the coastal polynya. The reduction in sea ice concentration results in increased heat loss to the atmosphere and thus an increase in convection and the formation of cold dense water in winter (Figure 13e and Movie S1). In the warm and cold regimes, the anomalies of sea ice concentration are much larger in summer than in winter (Figure S5 in Supporting Information S1), although it is the winter anomalies that are more important for the rate of cold water formation. Specifically in the warm regime, the sea ice concentration near the coast in the southern Bellingshausen Sea is reduced in both winter and summer in comparison to the cold regime (Figure S5 in Supporting Information S1). The cold regime has reduced wind speeds in Box C (Figure 12b), which will increase the sea ice concentration all year round (Figure 13b) and thus reduce heat loss to the atmosphere, convection, and wintertime formation of cold dense water. Within the coastal polynya (83°W, 73.5°S), periods of reduced sea ice concentrations in warm years (Figure 14a) allow for an on average deeper mixed layer depth (94 ± 21 m) and a deeper ventilation of cold, fresh surface waters (Figures 14b and 14c) that erodes the mCDW layer below. Periods of increased annual mean sea ice concentration in cold years (Figure 14a) show an on average shallower mixed layer depth (64 ± 20 m) and less deep ventilation of cold, fresh surface waters (Figures 14b and 14c). This reduced seasonality results in a slow build‐up of mCDW. Note that the location shown in Figure 14 is near the edge of increased sea ice concentration further to the west of Eltanin Bay in cold years (Figure 13b). Thus, increased salinity during the periods of cold, dense water formation in winter (Figure 14c) is related to brine rejection during sea ice formation. The gradual warming seen in the bottom temperatures (Figure 14d) is consistent with the net southward heat transport across 72°S into Eltanin and Latady Bays and positive air‐sea fluxes south of 72°S (ocean heat uptake) in cold years (Figures 10b and 10c). Deeper mixed layers during periods of reduced sea ice concentrations in warm years, compared to shallower mixed layers in cold years, is also consistent with the region north of the coastal polynya (83°W, 72.5°S, Figure S6 in Supporting Information S1). However, north of the coastal polynya, bottom temperatures demonstrate little to no seasonal variability and the transitions from warm to cold and cold to warm regimes are fairly abrupt (within a month).

The differences in sea ice cover, heat loss to the atmosphere, and subsequent formation of cold, dense water masses, provide a plausible explanation for the cooling of the water in Eltanin and Latady Bays in the warm regime. The net southward heat transport across 72°S and positive air‐sea fluxes south of 72°S in the cold regime provide a plausible explanation for the warming experienced in the cold years. Webber et al. (2017) presented observations of warming and cooling periods (spring 2009 and 2012) in Pine Island Bay in the Amundsen Sea. Although the years (2009 and 2012) from Webber et al. (2017) do not agree with the warming and cooling periods in the Bellingshausen Sea, the suggested mechanisms are consistent with our findings for the warm regime (cooling period, increased surface heat loss, deeper thermocline) and for the cold regime (warming period, decreased surface heat loss, shallower thermocline). In summary, our Bellingshausen Sea results imply a negative feedback mechanism that operates to maintain stable water mass temperatures in the long term: warming during the cold regime and cooling during the warm regime.

Dinniman et al. (2012) demonstrated using a simulation that the mixing over the WAP continental shelf in response to a stronger wind field removes more heat from the deeper shelf waters than the additional heat gained from an increased mCDW volume transport. Venables and Meredith (2014) showed using observations from 1994 to 2014 near Ryder Bay on the WAP that reduced sea ice concentration leads to increased mixing and heat loss in winter. These results on the WAP shelf are consistent with our results in the southern Bellingshausen Sea. The reduction in stratification persists into the following summer, preconditioning the water column for increased vertical mixing in the following winter so that more heat is mixed down in summer than was lost in winter. Our results in the southern Bellingshausen Sea suggest that during warm years the wind‐induced reduction of sea ice concentration in summer and winter leads to more cold, dense water formation in winter and increased ocean heat uptake in summer (Figure 14b). However, summer warming and winter cooling processes are much more variable in the warm regime than in the cold regime and do not provide a positive feedback loop suggested by Venables and Meredith (2014) on the WAP. Maintaining a long time series of year‐round observations on the Bellingshausen Sea continental shelf similar to that on the WAP would be a valuable contribution to the Southern Ocean Observing System (SOOS, www.soos.aq).

Narayanan et al. (2019) argued from seal‐acquired observations that in the years from 2004 to 2017 that there was no observable formation of dense shelf water in the Bellingshausen Sea. However, the density of seal data during winter months is sparse around the coast in Eltanin Bay, where convection and formation of cold dense water are found in GLORYS. Furthermore, the dense shelf water that Narayanan et al. (2019) referred to was defined as a salinity >34.5 and temperatures ≤−1.8 °C (Williams et al., 2016). This is more saline and colder than the cold dense water forming near the coast in Eltanin Bay in GLORYS, so the possibility remains that some water, colder, and denser than mCDW but not as cold and dense as the definition used by Narayanan et al. (2019) was formed in the Bellingshausen Sea during this time period. However, we also acknowledge that the lack of ice shelf cavities and the lack of in situ ocean observations for data assimilation into GLORYS may impact the properties of coastal water masses in GLORYS. The modeling study by St‐Laurent et al. (2015) in the Amundsen Sea demonstrated the importance of sea ice concentration and surface heat fluxes on warming and cooling periods, concluding that they directly impact ice shelf melt rates. The increased presence of buoyant ice shelf meltwater will also affect stratification in the region. In this study, we cannot confirm the impact on ice shelf melt rates and resulting water mass stratification in the Bellingshausen Sea as GLORYS does not include ice shelf cavities or ice shelf meltwater.

Overall, we have shown that changes in the ASL's location and intensity impact wind velocities, Ekman transports, SSH, and current structures in the Bellingshausen Sea region. Strong correlation coefficients of SSH (*r* ≈ ±0.65) and current speeds above 300 m (*r* ≈ −0.70) with the PC to support the significance of these findings (Figures 13c and 13d). We further demonstrate that the warm and cold regimes exhibit conditions that are linked to different tendencies of cooling and warming in association with wind‐induced changes of sea ice concentration in the southern Bellingshausen Sea.

## 5 Conclusions

In this study, we use the GLORYS12V1 reanalysis to study the temperature variability of waters below 300 m on the southern Bellingshausen Sea continental shelf over a period of 26 years from 1993 to 2018. The analysis of the first EOF mode and PC reveals a spatial pattern which demonstrates strongest temperature changes within Eltanin and Latady Bays, and a temporal pattern that allows a separation into warm and cold regimes.

Our results show that our definition of warm and cold years in the Bellingshausen Sea only partly agrees with observations (Jenkins et al., 2018; Webber et al., 2017) and simulations (Dotto et al., 2019; Dutrieux et al., 2014) in the adjacent Amundsen Sea. The first EOF mode (Figure 5) shows the opposite sign in the far west of the Bellingshausen Sea study region and agrees with the fact that the Amundsen Sea has a different variability pattern. Furthermore, our results show a negative feedback loop (warming in the cold regime, cooling in the warm regime) opposite to the positive feedback loop that Venables and Meredith (2014) demonstrated with observations near Ryder Bay on the WAP. This might indicate that due to spatial distance and differences in atmospheric forcing between the Bellingshausen, Amundsen Seas and the WAP, warm and cold periods are not in phase in these regions.

Our analysis of the conditions and processes occurring in the warm and cold regimes reveal that changes in the ASL's location and intensity impact wind velocities and Ekman transports in the Bellingshausen Sea region. The ASL is more intense and extends further east during the warm regime than during the cold regime. A consequence of the ASL extending less far east in the cold regime is that regions north of 72°S experience higher wind speeds (increase in east and southward wind components, Figures 12c and 12d). This increases the offshore Ekman transport and results in lower SSH on the Bellingshausen Sea continental shelf, where stronger SSH gradients above the continental slope and along the coast of Eltanin Bay amplify both the frontal jet and the Antarctic Coastal Current in the cold regime. Correlations with the PC confirm that the strongest relationship to the temperature variability below 300 m is found in the SSH (*r* ≈ ±0.65) and current speeds above 300 m in areas affected by the frontal jet (*r* ≈ −0.70). Importantly, the strong correlation between the PC and SSH (Figure 13c) suggest that satellite altimetry may be able to give a remote indication of warm and cold conditions in the Bellingshausen Sea, although note that SSH from satellite altimetry is not currently as high resolution as the SSH in GLORYS (Armitage et al., 2018; Auger et al., 2022).

**Figure 15:**
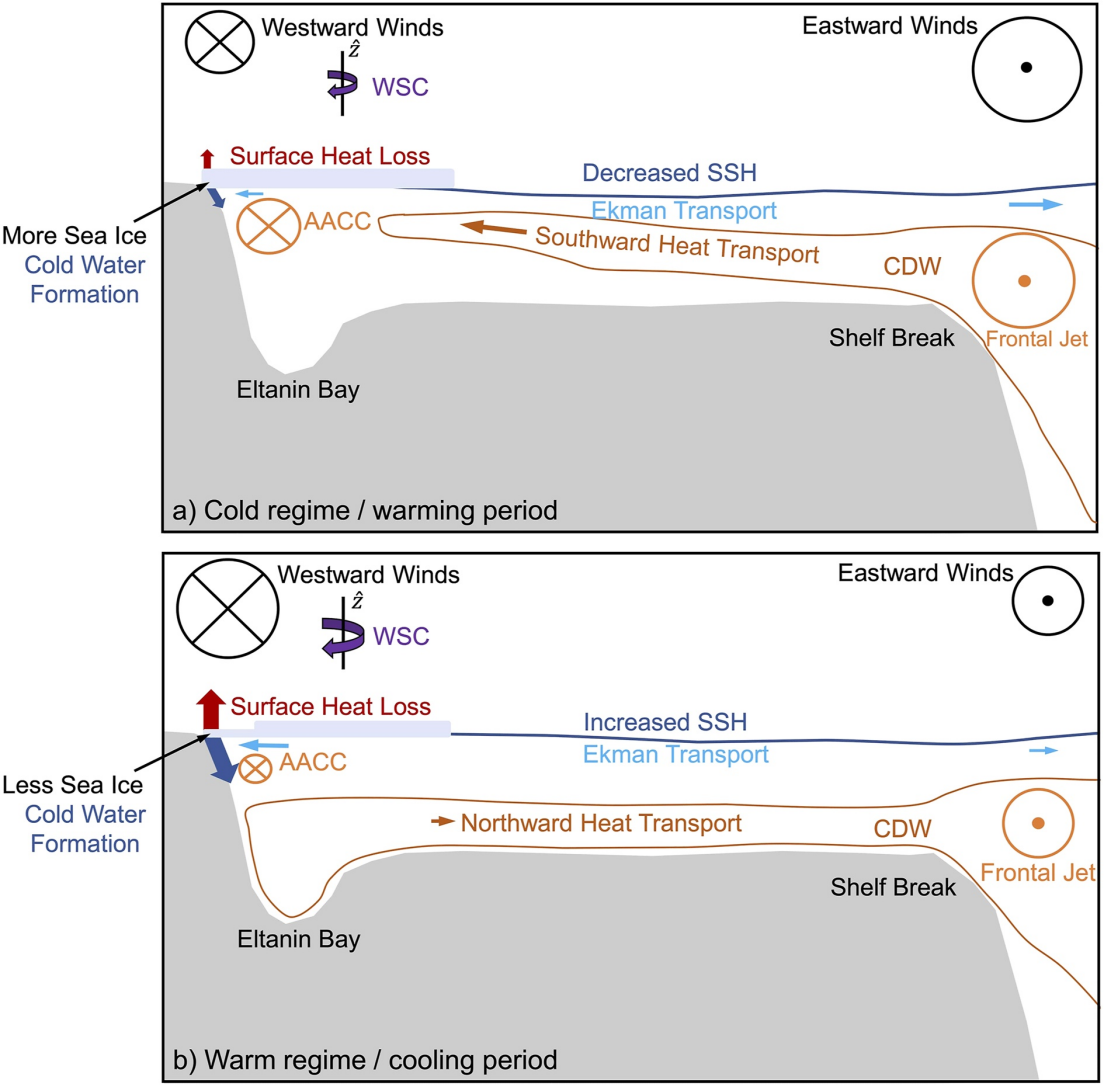
Schematics covering a meridional section from coast to shelf break presenting processes and conditions referred to in the main text during (a) the warming period in the cold regime and (b) cooling period in the warm regime. Wind stress curl is indicated with WSC.

The warm and cold regimes are also linked to different tendencies of cooling and warming (Figure 15). In the warm regime, a wind‐induced reduction of sea ice results in increased heat loss to the atmosphere that drives convection and the formation of cold dense water in winter, which is associated with a cooling of Eltanin and Latady Bays and a net northward heat transport. In contrast, increased sea ice conditions in the cold regime result in weakened heat loss to the atmosphere and a decrease in convection and formation of cold dense water in winter, which is associated with a gradual warming of Eltanin and Latady Bays and a net southward heat transport.

However, further reanalysis studies with a higher resolution bathymetry are needed to establish the southward heat transport through the Bellingshausen Sea's major troughs more accurately. A global reanalysis with a higher resolution temporal and spatial output is needed to analyze the effects of eddies and eddy heat fluxes in the Bellingshausen Sea region. Further inclusion of ice shelf cavities in a global reanalysis product is essential to include the analysis of processes such as ice shelf melting in future studies.

Climate model simulations indicate that the ASL will likely migrate poleward and eastward during the remainder of this century (Hosking et al., 2016), which will cause a southward migration of eastward winds (Holland et al., 2019) and lead to stronger eastward winds along the continental slope of the Amundsen and Bellingshausen Seas (Hosking et al., 2016). Stronger eastward winds above the continental slope would increase the offshore Ekman transport and result in even lower SSH on the Bellingshausen Sea continental shelf. Stronger SSH gradients above the shelf break and along the eastern and southern coasts of the Bellingshausen Sea would result in an intensification of both the frontal jet and the Antarctic Coastal Current. A southward migration of the ASL might also result in weakened winds in the southern Bellingshausen Sea, as the strong northwest winds would move further south over the continental land mass. This would lead to an increase of sea ice concentration in the southern Bellingshausen Sea, and thus a reduction in the heat loss to the atmosphere and a decrease in convection and cold water formation in winter. This would suggest a gradual warming of Eltanin and Latady Bays in the future.

## Supporting information

Supporting Information S1

Movie S1

## Data Availability

We are grateful to the originators of the many open‐access data sets synthesized in this study, the GLORYS12V1 reanalysis data (doi: https://doi.org/10.48670/moi-00021; Fernandez & Lellouche, 2021), the ERA5 data (doi: https://doi.org/10.24381/cds.f17050d7; Hersbach et al., 2019), and the R‐Topo2 data (doi: https://doi.org/10.1594/PANGAEA.856844; Schaffer et al., 2016).
