# Supplementary material for: Wind‐Induced Variability of Warm Water on the Southern Bellingshausen Sea Continental Shelf
Source: J Geophys Res Oceans. 2022 Nov 8;127(11):e2022JC018636. doi: 10.1029/2022JC018636 (PMC9787697; doi:10.1029/2022JC018636)
Supplement: Supplementary file 1 — Supporting Information S1 [file JGRC-127-e2022JC018636-s002.pdf]

Supporting Information for "Wind-induced Variability on the Southern Bellingshausen Sea Continental Shelf"

Ria Oelerich [1], Karen J. Heywood[1], Gillian M. Damerell[1] and Andrew F. Thompson[2]

[1]Centre for Ocean and Atmospheric Sciences, School of Environmental Sciences, University of East Anglia, Norwich, United Kingdom.

[2]Environmental Science and Engineering, California Institute of Technology, Pasadena, California

Additional Supporting Information (Files uploaded separately)

1. Captions for Movie S1

Contents of this file

1. Figures S2 to S6

Introduction. The supporting information includes one movie and four figures to accompany our manuscript. Movie S1 shows monthly means of sea ice fraction and sea surface heights (top panel), potential temperature (central panel) and practical salinity (bottom panel) along the meridional transect from 67-74°S at 83°W extracted from the GLORYS12V1 reanalysis in a time period from 1993-2018. Movie S1 is used to highlight that wind conditions in the warm regime (shown in the manuscript) are responsible for reduced sea ice concentration in the south of the meridional transect, where sea ice is more rapidly blown away from the coast to the north west. The reduction in sea ice

concentration results in increased heat loss to the atmosphere and thus an increase in convection and the formation of cold dense water in winter.

Figure S2 demonstrates the (a) summer and (b) winter long-term means of wind speeds extracted from ERA5 from 1993 to 2018. Figure S2 is used to show the long-term means for summer and winter, which the wind speed anomalies in Figure S3 and S4 are based on.

Figure S3 demonstrates the winter wind speed (a,b), zonal wind component (c,d) and meridional wind component (e,f) anomalies for the the warm and cold regimes defined in the manuscript. These anomalies are calculated using the winter long-term means shown in Figure S2. The anomalies are used to highlight the seasonality of wind speed and to highlight the differences in seasonality within the warm and cold regimes.

Figure S4 demonstrates the summer wind speed (a,b), zonal wind component (c,d) and meridional wind component (e,f) anomalies for the the warm and cold regimes defined in the manuscript. The anomalies are calculated using the summer long-term means shown in Figure S2. These anomalies are used to highlight the seasonality of wind speed and to highlight the differences in seasonality within the warm and cold regimes.

Figure S5 shows the (a,b) summer and (c,d) winter anomalies of sea ice concentration for the warm and cold regimes defined in the manuscript. The anomalies are calculated using the summer and winter long-term means shown in Figure 2e,f in the manuscript. These anomalies are used to highlight the seasonality of sea ice concentrations and to

highlight the differences in seasonality within the warm and cold regimes.

Figure S6 shows a monthly timeseries of (a) sea ice concentration, (b) potential temperature, (c) practical salinity and (d) bottom temperatures at 83°W and 72.5°S to show the seasonal variability and transitions from warm to cold and cold to warm regimes outside of the costal polynya region.

Movie S1. Monthly means of sea ice fraction and sea surface heights (top panel), potential temperature (central panel) and practical salinity (bottom panel) along the meridional transect from 67-74°S at 83°W extracted from the GLORYS12V1 Reanalysis in a time period from 1993-2018. Time (month and year) for each slide in the movie is given at the bottom left.

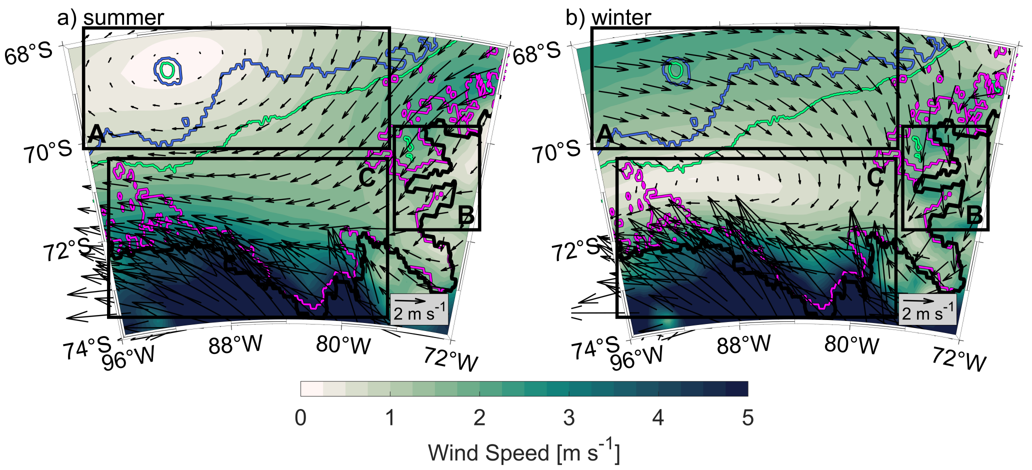

Figure S2. (a) Summer and (b) winter long term mean of wind speed from 1993 to 2018, extracted from ERA5 in the Bellingshausen Sea region, superimposed with velocity vectors for the (a) summer and (b) winter long term mean winds. Isobaths are coloured as in Fig. 2 in the manuscript. Boxes A, B and C highlight regions discussed in section 4 of the paper.

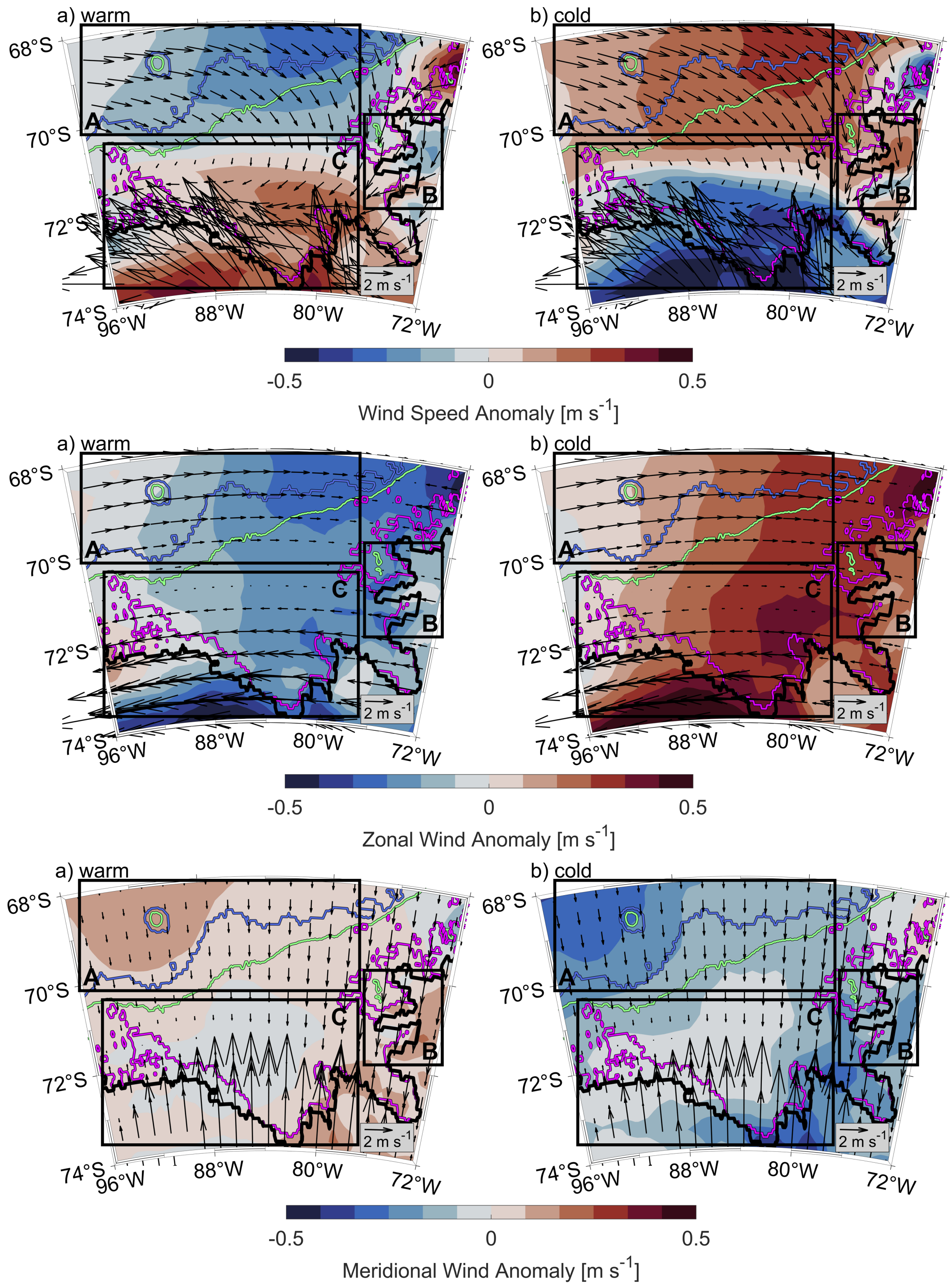

Figure S3. Anomalies from the winter long-term mean of (a,b) wind speed, (c,d) zonal wind component and (e,f) meridional wind component for the warm (a,c,e) and cold (b,d,f) regimes. The anomalies are calculated from winter long-term means as shown in Figure S2. Composite wind velocity vectors for the warm and cold regimes are superimposed. Boxes A, B and C highlight areas discussed in the text. Isobaths are coloured as in Fig. 2 of the manuscript.

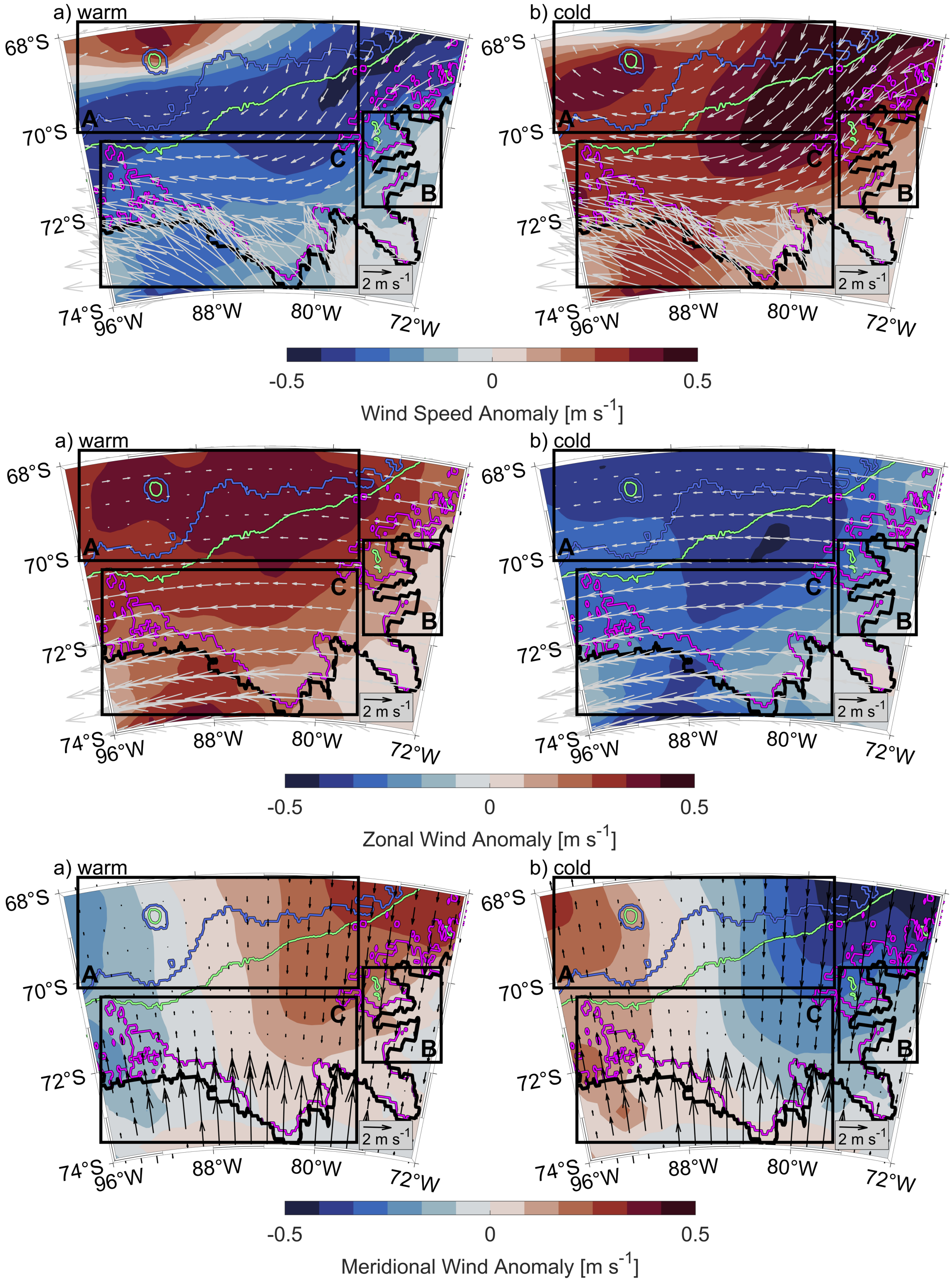

Figure S4. Anomalies from the summer long-term mean of (a,b) wind speed, (c,d) zonal wind component and (e,f) meridional wind component for the warm (a,c,e) and cold (b,d,f) regimes. The anomalies are calculated from summer long-term means as shown in Figure S2. Composite wind velocity vectors for the warm and cold regimes are superimposed. Boxes A, B and C highlight areas discussed in the text. Isobaths are coloured as in Fig. 2 of the manuscript.

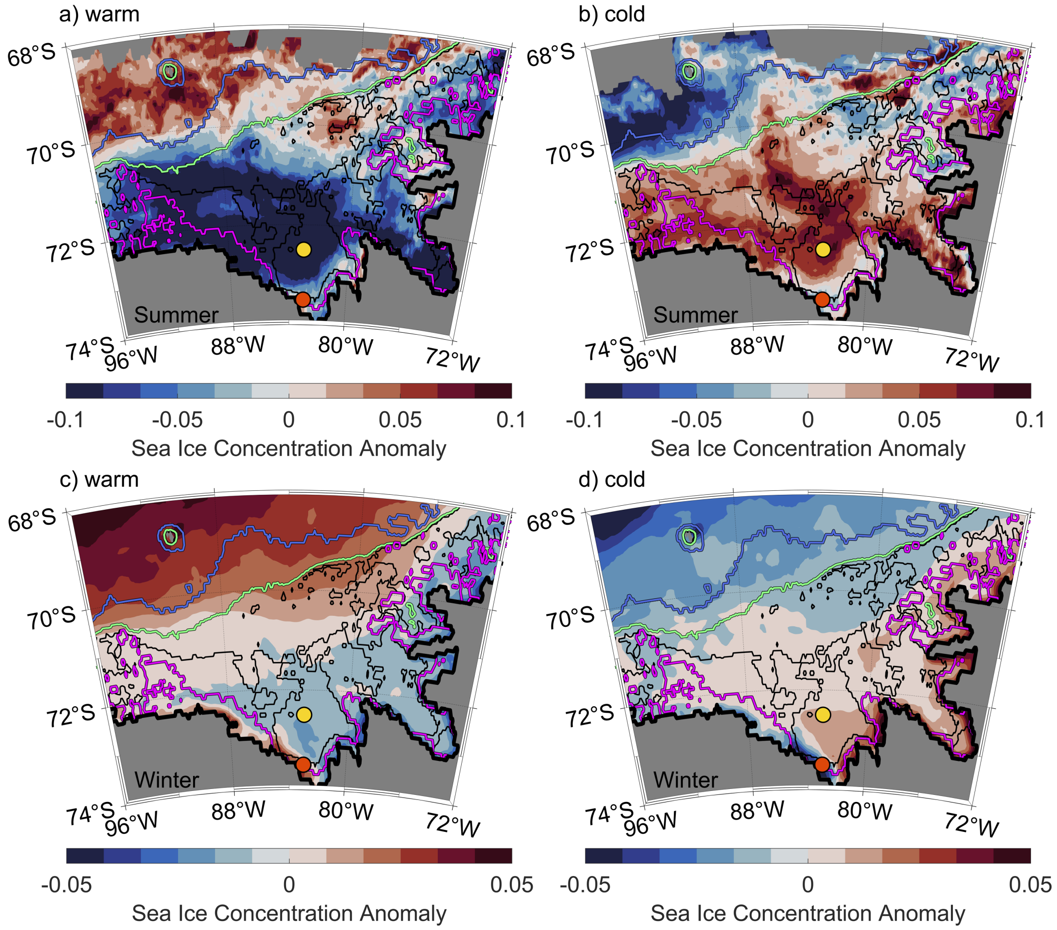

Figure S5. Sea ice anomalies for the summer (a,b) and winter (c,d) long-term means for (a,c) the warm regime and (b,d) the cold regime. The anomalies are calculated relative to the summer and winter long-term means as shown in Fig. 2e,f of the paper. Note the different colour scales used for summer and winter. Isobaths are coloured as in Fig. 1 of the paper, and the yellow and orange dots are as in Fig. 6.

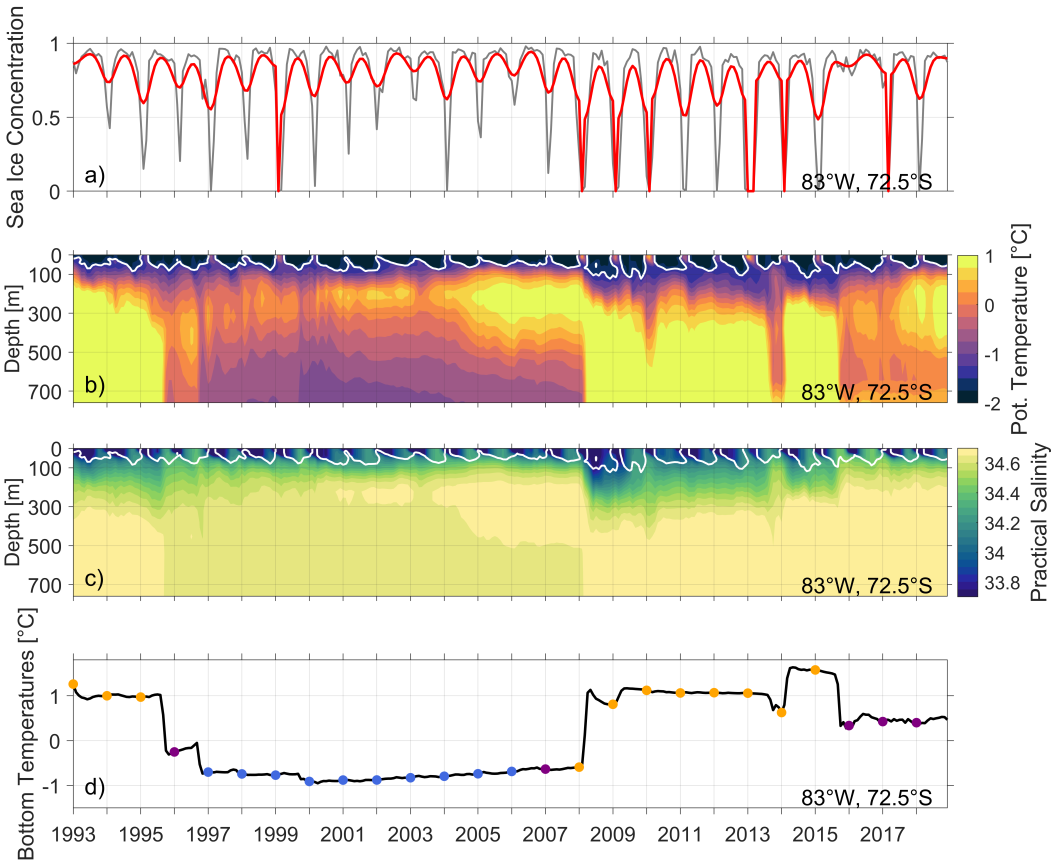

Figure S6. (a) Timeseries at 83°W, 72.5°S, showing: (a) monthly sea ice concentration (grey) and monthly sea ice concentration smoothed with a 12-month running mean (red), Hovmöller diagrams of (b) potential temperature and (c) practical salinity and (d) bottom temperatures. The white contour marks the -1.5°C isotherm associated with cold, dense water formation. The colored dots in (d) placed at the beginning of each year indicate which years are defined as warm regime, cold regime or transition years as defined in section 3 of the paper.
